# Radiotherapy plus cisplatin or cetuximab in low-risk human papillomavirus-positive oropharyngeal cancer (De-ESCALaTE HPV): an open-label randomised controlled phase 3 trial

**DOI:** 10.1016/S0140-6736(18)32752-1

**Published:** 2019-01-05

**Authors:** Hisham Mehanna, Max Robinson, Andrew Hartley, Anthony Kong, Bernadette Foran, Tessa Fulton-Lieuw, Matthew Dalby, Pankaj Mistry, Mehmet Sen, Lorcan O'Toole, Hoda Al Booz, Karen Dyker, Rafael Moleron, Stephen Whitaker, Sinead Brennan, Audrey Cook, Matthew Griffin, Eleanor Aynsley, Martin Rolles, Emma De Winton, Andrew Chan, Devraj Srinivasan, Ioanna Nixon, Joanne Grumett, C René Leemans, Jan Buter, Julia Henderson, Kevin Harrington, Christopher McConkey, Alastair Gray, Janet Dunn, De-ESCALaTE HPV Trial Group, Rafael Moleron, Orla McArdle, Karen Dyker, Hoda Al Booz, Lorcan O'Toole, Audrey Cook, David Husband, Vivienne Loo, Win Soe, Eleanor Aynsley, Thiagarajan Sridhar, Petra Jankowska, Mano Joseph, Konstantinos Geropantas, Deepali Vaidya, Matthew Griffin, Andrew Hartley, Rengarajan Vijayan, David Hwang, Kevin Harrington, Laura Pettit, Stephen Whitaker, Emma De Winton, Martin Rolles, Sinéad Brennan, Mehmet Sen, Ruheena Mendes, Martin Forster, Andrew Chan, Mererid Evans, Jan Buter, Devraj Srinivasan, Bernie Foran, Paul Nankivell, Jennifer Bryant, Neil Sharma, Rachel Spruce, Jill Brooks, Nikos Batis, Tom Roques, Margaret Bidmead, Huiqi Yang, Christopher Nutting, Justine Tyler, Julia Henderson, Helen Baines, Anne Gasnier, Elizabeth Miles, Catharine Clark, Mererid Evans

**Affiliations:** a Institute for Head and Neck Studies and Education (InHANSE), University of Birmingham, Birmingham, UK; b Newcastle University, Newcastle, UK; c University Hospitals Birmingham, Birmingham, UK; d Weston Park Hospital, Sheffield, UK; e University of Warwick, Coventry, UK; f St James's Institute of Oncology, Leeds, UK; g Queen's Centre for Oncology, Castle Hill Hospital, Cottingham, UK; h Bristol Haematology and Oncology Centre, Bristol, UK; i St James' University Hospital, Leeds, UK; j Aberdeen Royal Infirmary, Aberdeen, UK; k Royal Surrey County Hospital, Surrey, UK; l St Luke's Hospital, Cancer Trials Ireland, and St Luke's Institute of Cancer Research, Dublin, Ireland; m Cheltenham General Hospital, Cheltenham, UK; n Nottingham University Hospitals NHS Trust, Nottingham, UK; o James Cook University Hospital, Middlesbrough, UK; p Abertawe Bro Morgannwg University Health Board, Port Talbot, UK; q Royal United Hospitals Bath NHS Foundation Trust, Bath, UK; r University Hospital Coventry and Warwickshire NHS Trust, Coventry, UK; s Western General Hospital, Edinburgh, UK; t Beatson West of Scotland Cancer Centre, Glasgow, UK; u VU University Medical Centre, Amsterdam, Netherlands; v National RTTQA Group, Royal Marsden Hospital, London, UK; w Institute of Cancer Research and Royal Marsden Hospital, London UK; x University of Oxford, Oxford, UK

## Abstract

**Background:**

The incidence of human papillomavirus (HPV)-positive oropharyngeal cancer, a disease affecting younger patients, is rapidly increasing. Cetuximab, an epidermal growth factor receptor inhibitor, has been proposed for treatment de-escalation in this setting to reduce the toxicity of standard cisplatin treatment, but no randomised evidence exists for the efficacy of this strategy.

**Methods:**

We did an open-label randomised controlled phase 3 trial at 32 head and neck treatment centres in Ireland, the Netherlands, and the UK, in patients aged 18 years or older with HPV-positive low-risk oropharyngeal cancer (non-smokers or lifetime smokers with a smoking history of <10 pack-years). Eligible patients were randomly assigned (1:1) to receive, in addition to radiotherapy (70 Gy in 35 fractions), either intravenous cisplatin (100 mg/m^2^ on days 1, 22, and 43 of radiotherapy) or intravenous cetuximab (400 mg/m^2^ loading dose followed by seven weekly infusions of 250 mg/m^2^). The primary outcome was overall severe (grade 3–5) toxicity events at 24 months from the end of treatment. The primary outcome was assessed by intention-to-treat and per-protocol analyses. This trial is registered with the ISRCTN registry, number ISRCTN33522080.

**Findings:**

Between Nov 12, 2012, and Oct 1, 2016, 334 patients were recruited (166 in the cisplatin group and 168 in the cetuximab group). Overall (acute and late) severe (grade 3–5) toxicity did not differ significantly between treatment groups at 24 months (mean number of events per patient 4·8 [95% CI 4·2–5·4] with cisplatin *vs* 4·8 [4·2–5·4] with cetuximab; p=0·98). At 24 months, overall all-grade toxicity did not differ significantly either (mean number of events per patient 29·2 [95% CI 27·3–31·0] with cisplatin *vs* 30·1 [28·3–31·9] with cetuximab; p=0·49). However, there was a significant difference between cisplatin and cetuximab in 2-year overall survival (97·5% *vs* 89·4%, hazard ratio 5·0 [95% CI 1·7–14·7]; p=0·001) and 2-year recurrence (6·0% *vs* 16·1%, 3·4 [1·6–7·2]; p=0·0007).

**Interpretation:**

Compared with the standard cisplatin regimen, cetuximab showed no benefit in terms of reduced toxicity, but instead showed significant detriment in terms of tumour control. Cisplatin and radiotherapy should be used as the standard of care for HPV-positive low-risk patients who are able to tolerate cisplatin.

**Funding:**

Cancer Research UK.

## Introduction

The incidence of oropharyngeal squamous cell carcinoma is increasing rapidly in high-income countries,^1^, ^2^ reaching epidemic proportions in some estimates.^3^ This increase has been attributed to a rise in human papillomavirus (HPV) infection. HPV-positive oropharyngeal squamous cell carcinoma is considered to be a distinct disease entity from HPV-negative head and neck cancer. The disease affects younger adults and treatment can be successful.^4^ HPV status, tumour nodal stage, and smoking history define three groups with distinct survival outcomes: low risk (HPV-positive, <10 pack-years; 3-year overall survival 93%), intermediate risk (HPV-positive, >10 pack-years; 3-year overall survival 71%) and high risk (HPV-negative; 3-year overall survival 48%).^4^

Cisplatin-based chemoradiotherapy and cetuximab bioradiotherapy are both approved by the US Food and Drug Administration for treatment of head and neck cancer, with cisplatin being standard of care for advanced oropharyngeal squamous cell carcinoma in most countries. However, concurrent cisplatin therapy is associated with substantial increases in acute, sometimes life-threatening, toxicity,^5^, ^6^, ^7^ compared with radiotherapy alone. The treatment also increases long-term sequelae,^5^, ^7^, ^8^ including xerostomia and dysphagia. Consequently, young patients with HPV-positive oropharyngeal squamous cell carcinoma might endure substantial, life-changing side-effects of treatment, that could affect their quality of life, for several decades.

**Research in context**

**Evidence before this study**

Standard treatment for human papillomavirus (HPV)-positive oropharyngeal cancer (cisplatin-based chemoradiotherapy) results in considerable acute and long-term toxicity. Wide consensus exists about the need for de-escalation treatments with decreased toxicity and similar survival. Cetuximab, an epidermal growth factor receptor inhibitor approved by the US Food and Drug Administration, is thought to result in reduced toxicity and thus might represent an ideal form of de-escalation in this setting. Meta-analyses of existing small, mainly retrospective studies reported poorer outcomes with cetuximab than with cisplatin for head and neck cancers overall, but a possible benefit in HPV-positive oropharyngeal cancer.

**Added value of this study**

Results of our open-label randomised controlled phase 3 trial show that, in patients with low-risk HPV-positive oropharyngeal cancer, not only did cetuximab result in similar rates of severe and all-grade toxicity to cisplatin but it importantly resulted in poorer overall survival and higher rates of locoregional recurrence and distant metastases than did standard cisplatin therapy.

**Implications of all the available evidence**

Concomitant cisplatin and radiotherapy should remain the standard of care for patients with low-risk HPV-positive oropharyngeal cancer. Our findings also suggest caution with de-escalation strategies and highlight the importance of phase 3 trial data before changing clinical practice.

There is global consensus about the need for treatment de-escalation (reduction of toxicity while preserving anti-tumour efficacy) for these patients.^9^ One such strategy seeks to substitute cetuximab for cisplatin as the radiosensitiser. Radiotherapy can induce epidermal growth factor receptor (EGFR) expression in head and neck cancers, resulting in acquired resistance.^10^ Cetuximab, a targeted EGFR inhibitor, might help overcome this resistance and might also induce antibody-dependent cell-mediated cytotoxicity. In a randomised trial,^11^ bioradiotherapy with cetuximab was shown to significantly improve overall survival compared with radiotherapy alone (median 49·0 months *vs* 29·3 months; hazard ratio [HR] 0·74; p=0·03) in patients with head and neck cancer, and, in an updated analysis,^12^ in patients with HPV-positive oropharyngeal squamous cell carcinoma (HR 0·16 [95% CI 0·07–0·36]). Since toxicity rates (except for rash) were broadly similar between the two groups, cetuximab could potentially represent a less toxic alternative to cisplatin in this setting.

Conversely, an inverse association between HPV positivity and EGFR status has been reported.^13^ Therefore, EGFR inhibition might not be as effective as chemotherapy in HPV-positive oropharyngeal squamous cell carcinoma. Studies have reported conflicting results in this regard^14^, ^15^ and, as yet, no randomised comparisons have been done in HPV-positive patients. The De-ESCALaTE trial aimed to compare the toxicity, survival, and time-to-recurrence outcomes of cetuximab versus cisplatin in patients with low-risk, HPV-positive oropharyngeal squamous cell carcinoma receiving radiotherapy in the curative setting. As these treatments might have different effects on quality of life, especially swallowing, relevant patient-reported outcomes were also measured between the two groups.

## Methods

### Study design

This open-label randomised controlled phase 3 trial was done at 32 head and neck treatment centres in Ireland (n=1), the Netherlands (n=1), and the UK (n=30).

The first and last authors and the Trial Management Group designed the study, which was coordinated by Warwick Clinical Trials Unit. Sample collection and coordination was done by the University of Birmingham, UK, and p16 immunohistochemistry was done at Newcastle University, UK. The authors vouch for the accuracy and completeness of the data and analysis, and for adherence to the study protocol. All authors contributed to the writing of the manuscript. The study protocol is available online.

All treating hospitals were approved as head and neck treatment centres by their country's health authorities. All centres and oncologists completed the trial's central radiotherapy quality assurance accreditation (see appendix for more information).

### Patients

Eligible patients were recruited by their treating clinicians. Patients had to be aged at least 18 years with a histologically confirmed diagnosis of advanced oropharyngeal squamous cell carcinoma (American Joint Committee on Cancer/International Union for Cancer Control [AJCC/UICC] tumour, node, and metastasis [TNM] 7th Edition manual: T3N0–T4N0, and T1N1–T4N3) that was classified as low risk as per the Ang classification:^4^ that is, the tumour sample had to be positive on p16 immunohistochemistry, and the patient had to be a non-smoker or have a lifetime self-reported smoking history of less than 10 pack-years. Patients had to have an Eastern Cooperative Oncology Group (ECOG) performance status score of 0 or 1, and adequate renal, haematological, and hepatic function for cisplatin-based curative chemoradiotherapy.

Formalin-fixed, paraffin-embedded tumour samples were histologically confirmed to be squamous cell carcinoma and tested in two quality-assured, central laboratories for p16 by immunohistochemistry, by use of proprietary reagents (CINtec Histology kit; Roche mtm labsAG; Basel, Switzerland). p16 was scored positive if 70% or more of malignant cells showed strong and diffuse nuclear and cytoplasmic staining.^16^ High-risk HPV DNA in-situ hybridisation was done with proprietary reagents (INFORM-HPV-III Family 16 Probe-B, Ventana Medical Systems Inc, Tuscon, USA). The probe cocktail detects HPV genotypes 16, 18, 31, 33, 35, 45, 52, 56, 58, and 66, and is visualised as a blue reaction product in malignant cells.^17^ Patients reported smoking history and alcohol consumption by self-completed questionnaires, and their comorbidities were graded by the recruiting clinician. Patients were excluded if they had T1–T2N0 disease or were classified as HPV-negative, high-risk, or HPV-positive oropharyngeal squamous cell carcinoma intermediate-risk on the Ang classification. The study was approved by the Coventry and Warwickshire Research Ethics Committee. Patients were recruited by their treating clinicians and all patients provided written informed consent.

### Randomisation and masking

Eligible patients underwent computer-generated central randomisation. Patients were randomly assigned in a 1:1 ratio to receive cisplatin-based chemoradiotherapy or cetuximab bioradiotherapy. Trial-group assignments were balanced by use of a bespoke minimisation algorithm according to centre, tumour stage (TNM7: T1–T2 *vs* T3–T4), nodal stage (N0–1 *vs* N2–3), radiotherapy site (unilateral; bilateral), and planned gastrostomy insertion before treatment.

### Procedures

Eligible patients were randomly assigned to undergo intensity-modulated radiotherapy with either three doses of intravenous cisplatin 100 mg/m^2^ on days 1, 22, and 43 of radiotherapy or intravenous cetuximab 400 mg/m^2^ loading dose 1 week before followed by seven weekly infusions of 250 mg/m^2^ during radiotherapy. Patients were assessed for treatment response 12 weeks after radiotherapy completion by clinical examination and by CT, MRI, or PET-CT scan. Follow-up consisted of clinical examination, monthly in the first year and every 2 months in the second year, for at least 24 months after treatment completion.

### Outcomes

The primary outcome was overall (acute and late) severe toxicity (grades 3–5). Treatment toxicity was assessed with the Common Toxicity Criteria Adverse Events (CTCAE), version 4, for a period of 24 months from the end of treatment. Secondary outcomes were overall survival, time to recurrence, quality of life, swallowing, and acute and late severe toxicities reported separately; suspected recurrences were assessed by imaging and biopsy. Patients completed the paper-based European Organisation for Research and Treatment of Cancer Quality-of-Life Questionnaire–Core 30 general (EORTC QLQ-C30, version 3) questionnaire and the EORTC QLQ-H&N35 questionnaire specific to head and neck cancers,^18^ and the M.D. Anderson Dysphagia Inventory^19^ at baseline before treatment, at radiotherapy completion, and then at 3, 6, 12, and 24 months after treatment.

### Statistical analysis

A target sample size of 304 patients (152 in each group) was calculated to enable detection of reductions greater than 25% in the overall number of severe (grade 3–5) acute and late toxicities with a two-sided test at the 5% level of significance, allowing for 10% dropout with greater than 90% power, assuming an average of 2·5 overall severe events per patient.

Recruitment of 304 patients was also estimated to allow detection of a 50% reduction in late severe toxicities with at least 90% power and a 25% or greater reduction in acute severe toxicities with 85% power. An additional 30 patients were recruited to allow for withdrawals and any higher risk (T4,N3) patients recruited. The interim analysis was done after the first 200 patients were recruited. More details are provided in the appendix.

An intention-to-treat analysis was done for all outcomes, and a per-protocol analysis was done for primary outcomes and secondary outcomes of toxicity and survival. All patients allocated to treatment groups were included in the intention-to-treat analysis, even if they did not receive the treatment. Patients who withdrew or who had major protocol violations as assessed by the independent trial monitoring team were excluded from the per-protocol analyses. All analyses were unblinded.

Mean numbers per patient of toxicity events (short-term [acute] toxicity and adverse long-term [late] effects, based on the TAME method of reporting toxicities^6^) were compared by *t* tests. Proportions of patients affected by one or more toxicity event were compared by Pearson's χ^2^ test. A severe toxic event was defined as a toxicity assessed as grade 3–5 by CTCAE, version 4. The type of event was characterised by the CTCAE, version 4, System Organ Class and Term. Toxicities were classified as acute if they first appeared during or up to 3 months after treatment, and were classified as late if they persisted, or first appeared, more than 3 months up to 24 months after treatment. Multiple occurrences of events of a single toxicity type within an analysis time period are counted as a single event. Events that were present both within 90 days after treatment and remained after that period were counted as acute events and also as late events but were not counted twice when analysing the overall number of acute and late events. Toxicities reported as part of a serious adverse event notification but that were not reported as a toxicity event were added to the counts of toxicity events.

Overall survival and time to recurrence were measured from the date of randomisation and compared by the log-rank test with all-cause mortality in the intention-to-treat population.

Deaths were classified as being due to head and neck cancer or to other causes. Recurrences could be loco-regional or distant, or both. Patients who died from head and neck cancer causes as the first event were classed as recurrences. New primaries and persistent nodal disease (detected within 90 days from randomisation) were not included in the analysis of time to recurrence. Patients on follow-up and patients lost to follow-up were censored at the last date at which they were known to be alive. 95% CIs were generated with a univariate Cox proportional hazards model. The proportional assumption was tested by plotting the observed Kaplan-Meier values against the Cox predicted values. The effect of randomised treatment on outcome was also assessed after adjusting for known prognostic factors with a multivariate Cox proportional hazards model. Two post-hoc subgroup sensitivity analyses examined the association with overall survival of the very low-risk group with TNM-8 stage I and II disease, and those who were doubly positive for p16 and HPV-DNA in-situ hybridisation.

Standard scoring methods were applied to quality-of-life questionnaires.^18^ Missing quality-of-life scores were not imputed. All scores were normalised, ranging from 0 to 100, and transformed to unweighted summated scales in which higher scores indicated better health. Global quality of life was assessed with the EORTC QLQ C30 global measure. Separate comparisons were made at each timepoint. Unadjusted p values were used. On the EORTC questionnaires and the M.D. Anderson Dysphagia Inventory, a 10-point difference in scores was considered to be clinically relevant.^19^, ^20^ Analyses were done with Stata, version 15.1.

This trial is registered with the ISRCTN registry, number ISRCTN33522080.

### Role of the funding source

The funder of the study had no role in study design, data collection, data analysis, data interpretation, or writing of the report. The corresponding author had full access to all the data in the study and had final responsibility for the decision to submit for publication.

## Results

**Figure 1:**
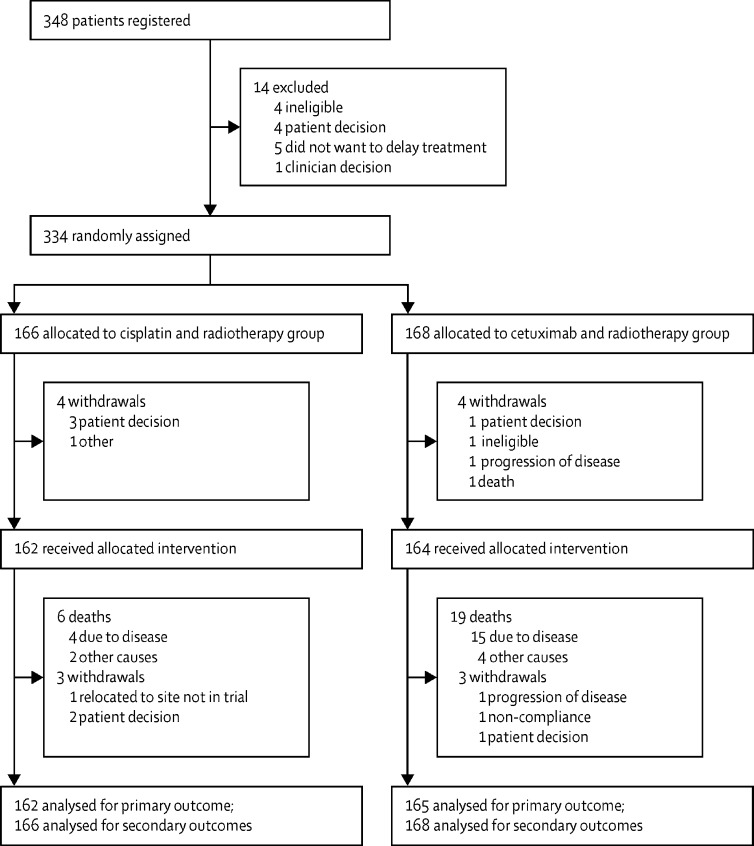
Trial profile

Between Nov 12, 2012, and Oct 1, 2016, 334 patients were recruited (166 in the cisplatin group and 168 in the cetuximab group). There were seven withdrawals from the treatment protocol before treatment started and six after starting treatment (figure 1; appendix). All patients allocated to treatment groups were included in the intention-to-treat analyses, and 159 patients in the cisplatin group and 162 in the cetuximab group were included in the per-protocol analyses. There was no crossover between groups.

We observed no notable imbalances in baseline characteristics (age, tumour site, tumour stage, smoking history, performance status, and comorbidities) between the two groups (table 1; appendix). The mean age was 57 years. 80% of patients were men, 65% had T1–T2 disease (TNM 7), 76% had N2–N3 disease (TNM 7), and 46% were either current or past smokers, with a median lifetime smoking history of 8 pack-years (table 1). Of the 324 (97%) patients tested for HPV-DNA on in-situ hybridisation, 304 (94%) were positive, 20 were negative, and ten were unknown. At the 3-month post-treatment assessment, 165 (51%) patients were assessed with PET-CT and 162 (49%) by MRI, with equal distribution among the groups.

**Table 1:** Baseline characteristics of patients

	**Cisplatin group (n=166)**	**Cetuximab group (n=168)**	**All patients (n=334)**
**Age, years**			
Mean	57·0 (7·8)	57·0 (8·3)	57·0 (8·0)
Median	56·5 (52·0–62·0)	57·0 (51·0–64·0)	57·0 (52·0–63·0)
**Sex**			
Men	132 (80%)	134 (80%)	266 (80%)
Women	34 (20%)	34 (20%)	68 (20%)
**HPV testing results (n=324)**			
p16-positive, HPV-ISH positive	151 (94%)	153 (94%)	304 (94%)
p16-positive, HPV-ISH negative	10 (6%)	10 (6%)	20 (6%)
**Tumour stage (TNM 7)**			
T1–T2	109 (66%)	107 (64%)	216 (65%)
T3–T4	57 (34%)	61 (36%)	118 (35%)
T4 only	32 (19%)	24 (14%)	56 (17%)
**Nodal stage (TNM 7)**			
N0–N1	40 (24%)	41 (24%)	81 (24%)
N2–N3	126 (76%)	127 (76%)	253 (76%)
N3 only	1 (1%)	1 (1%)	2 (1%)
**Primary tumour laterality (n=328)**			
Left only	80 (49%)	86 (52%)	166 (51%)
Right only	75 (46%)	67 (41%)	142 (43%)
Midline or any combination	8 (5%)	12 (7%)	20 (6%)
**Primary subsite (n=329)**			
Base of tongue	54 (33%)	58 (35%)	112 (34%)
Tonsil	107 (65%)	104 (63%)	211 (64%)
Other	3 (2%)	3 (2%)	6 (2%)
**ECOG performance status (n=328)**			
0	142 (87%)	149 (91%)	291 (89%)
1	22 (13%)	15 (9%)	37 (11%)
**Current alcohol consumption (n=329)**			
No	44 (27%)	37 (22%)	81 (25%)
Yes	120 (73%)	128 (78%)	248 (75%)
Median reported units per week	10·0 (4·0–20·0)	10·0 (4·0–20·0)	10·0 (4·0–20·0)
**Ever smoked?**			
No	94 (57%)	86 (51%)	180 (54%)
Yes	72 (43%)	82 (49%)	154 (46%)
Median pack years	6·5 (3·0–13·0)	8·0 (3·0–15·0)	8·0 (3·0–15·0)
**Radiotherapy**			
Unilateral	34 (20%)	34 (20%)	68 (20%)
Bilateral	132 (80%)	134 (80%)	266 (80%)
**Planned PEG use before treatment**			
No	57 (34%)	58 (34%)	115 (34%)
Yes	109 (66%)	110 (66%)	219 (66%)

Data are n (%), mean (SD), or median (IQR). There were no significant differences between the two treatment groups for any of the factors. Continuous variables were compared with *t* tests or Mann-Whitney U tests, and categorical variables compared with χ^2^ test. HPV=human papillomavirus. ISH=in-situ hybridisation. TNM=tumour, node, and metastasis. ECOG=Eastern Cooperative Oncology Group. PEG=percutaneous endoscopic gastrostomy.

The median duration from randomisation to start of radiotherapy was 14 days (IQR 11–17) and did not differ between groups. Only eight (5%) patients in the cisplatin group and four (3%) in the cetuximab group received a dose of less than 70 Gy (p=0·256; appendix), all patients received a dose of 65 Gy or more, and 332 (99%) received intensity-modulated radiation therapy. Radiotherapy interruptions or modifications occurred in 12 (9%) patients receiving cisplatin and 14 (7%) receiving cetuximab.

In the cisplatin group, 62 (38%) patients received all three cycles of cisplatin, 83 (51%) received two cycles, and 16 (10%) received one cycle. Of those who received one or two cycles, nine received one or two doses of carboplatin instead (appendix). One patient did not receive any chemotherapy because of sepsis. The median total cisplatin dose received was 200 mg/m^2^ (IQR 200–300) and 26 (16%) received less than 200 mg/m^2^ in total. The main reasons for discontinuation or reduction in cisplatin dose were myelosuppression, oral or gastrointestinal toxicity, or nausea and vomiting.

In the cetuximab group, 130 (79%) patients received all eight cycles of cetuximab; 23 (14%) received seven cycles, mainly omitting the final dose (appendix). The median total cetuximab dose received was 2150 mg/m^2^ (IQR 2133–2150). The main reasons for discontinuation were skin rash, patient decision, and oral or gastrointestinal toxicity.

Patients had a median follow up of 25·9 months (95% CI 25·5–26·0). The primary outcome of reported overall severe (grades 3–5) toxicity did not differ significantly between treatment groups; the mean number of events per patient was 4·8 (95% CI 4·2–5·4) for cisplatin and 4·8 (4·2–5·4) for cetuximab (p=0·98). Overall toxicity of all grades did not differ significantly either; the mean number of events per patient was 29·2 (95% CI 27·3–31·0) in the cisplatin group versus 30·1 (28·3–31·9) in the cetuximab group (p=0·49; table 2; appendix).

**Table 2:** Mean number of acute, late, and overall toxicity events per patient, by treatment group

		**Cisplatin plus radiotherapy (95% CI)**	**Cetuximab plus radiotherapy (95% CI)**	**p value**
**Primary outcome**				
Overall				
	Grade 3–5	4·81 (4·23–5·40)	4·82 (4·22–5·43)	0·98
	All grades	29·15 (27·33–30·97)	30·05 (28·26–31·85)	0·49
**Secondary outcomes**				
Acute short-term toxicities				
	Grade 3–5	4·43 (3·88–4·97)	4·35 (3·84–4·86)	0·84
	All grades	19·96 (18·81–21·12)	20·35 (19·18–21·52)	0·64
Severe late toxicities				
	Grade 3–5	0·41 (0·29–0·54)	0·48 (0·30–0·67)	0·53
	All grades	9·44 (8·53–10·34)	9·87 (9·02–10·72)	0·49

*t* test used to compare treatment groups. No adjustments have been made for multiple testing. Toxicity assessed with Common Toxicity Criteria for Adverse Events, version 4.0.

In the acute period, severe short-term toxicities did not differ significantly between groups; the mean number of events per patient was 4·4 (95% CI 3·9–4·97) for the cisplatin group versus 4·4 (3·8–4·9) for the cetuximab group (p=0·84). Rates of all-grade toxicity did not differ significantly either; the mean number of events per patient was 20·0 (95% CI 18·8–21·1) in the cisplatin group versus 20·4 (19·2–21·5) in the cetuximab group (p=0·64; table 2).

Severe late toxicity events (adverse long-term [late] events) did not differ significantly between the cisplatin and cetuximab groups (mean 0·4 *vs* 0·5 events per patient; p=0·53), and neither did all-grade late toxicity events (mean 9·4 *vs* 9·9 events per patient; p=0·49; table 2 appendix). The proportions of patients affected by either overall severe (grade 3–5) or all grade toxicities also did not differ significantly between the two groups (appendix).

Similar results were seen for all these outcomes in the per-protocol population (appendix). Furthermore, a post-hoc subgroup analysis of toxicity outcomes in patients who received the complete treatment regimens (three doses of cisplatin or eight doses of cetuximab) showed similar outcomes to the intention-to-treat analysis, with no significant differences between the two groups in any of the primary or secondary toxicity outcomes (appendix).

The range of toxicities differed between the two treatment groups (table 3). For cisplatin, the most common acute severe toxicities were gastrointestinal (mean 2·12 events per patient) and the most common late toxicities were gastrointestinal (mean 0·2 events per patient) and labyrinthine (otological symptoms including hearing loss, tinnitus, and vertigo; mean 0·1 events per patient). Cisplatin also caused more haematological, metabolic, and renal toxicity than did cetuximab. For cetuximab, the most common severe toxicities were also gastrointestinal (mean 1·9 acute and 0·2 late events per patient). Cetuximab also caused more skin toxicity and infusion reactions in the acute phase than did cisplatin (table 3).

**Table 3:** Range of acute, late, and overall (combined) severe toxicities, by type, mean number of events per patient, and proportion of patients affected by one or more toxicity, by treatment group

	**Acute severe toxicity**				**Late severe toxicity**				**Overall severe toxicity**			
	Cisplatin plus radiotherapy (n=162)		Cetuximab plus radiotherapy (n=165)		Cisplatin plus radiotherapy (n=162)		Cetuximab plus radiotherapy (n=165)		Cisplatin plus radiotherapy (n=162)		Cetuximab plus radiotherapy (n=165)	
	Mean events	Patients	Mean events	Patients	Mean events	Patients	Mean events	Patients	Mean events	Patients	Mean events	Patients
Blood and lymphatic system disorders	0·13	20 (12%)	0·01	2 (1%)	0·01	1 (1%)	0·01	2 (1%)	0·14	21 (13%)	0·02	4 (2%)
Cardiac disorders	0·01	2 (1%)	..	0	..	0	..	0	0·01	2 (1%)	..	0
Ear and labyrinth disorders	0·02	3 (2%)	0·04	4 (2%)	0·14	21 (13%)	0·05	8 (5%)	0·15	24 (15%)	0·08	12 (7%)
Gastrointestinal disorders	2·12	130 (80%)	1·88	129 (78%)	0·15	19 (12%)	0·21	23 (14%)	2·25	147 (91%)	2·09	151 (92%)
General disorders and administration site	0·20	31 (19%)	0·18	27 (16%)	0·01	2 (1%)	0·02	2 (1%)	0·22	33 (20%)	0·21	29 (18%)
Infections and infestations	0·16	19 (12%)	0·17	21 (13%)	0·01	1 (1%)	0·02	2 (1%)	0·16	19 (12%)	0·19	23 (14%)
Injury, poisoning, and procedural complications	0·16	26 (16%)	0·39	64 (39%)	..	0	0·01	1 (1%)	0·16	26 (16%)	0·39	65 (39%)
Investigations	0·08	11 (7%)	0·04	7 (4%)	0·01	2 (1%)	0·01	2 (1%)	0·09	13 (8%)	0·05	9 (6%)
Metabolism and nutrition disorders	0·61	77 (48%)	0·45	65 (39%)	0·02	4 (3%)	0·04	7 (4%)	0·64	81 (50%)	0·49	72 (44%)
Musculoskeletal and connective tissue disorders	0·07	10 (6%)	0·10	14 (9%)	0·02	4 (3%)	0·03	5 (3%)	0·09	13 (8%)	0·13	19 (12%)
Nervous system disorders	0·06	9 (6%)	0·10	16 (10%)	0·01	1 (1%)	0·02	3 (2%)	0·06	10 (6%)	0·12	19 (12%)
Psychiatric disorders	0·06	9 (6%)	0·04	7 (4%)	..	0	0·01	1 (1%)	0·06	9 (6%)	0·05	8 (5%)
Renal and urinary disorders	0·07	11 (7%)	..	0 (0%)	0·01	2 (1%)	0·01	1 (1%)	0·09	13 (8%)	0·01	1 (1%)
Respiratory, thoracic, and mediastinal disorders	0·60	78 (48%)	0·50	70 (42%)	0·02	3 (2%)	0·03	5 (3%)	0·62	81 (50%)	0·52	74 (45%)
Skin and subcutaneous tissue disorders	0·06	7 (4%)	0·42	50 (30%)	..	0	0·01	1 (1%)	0·06	7 (4%)	0·43	51 (31%)
Vascular disorders	0·02	4 (3%)	0·02	4 (2%)	0·01	1 (1%)	0·01	2 (1%)	0·03	5 (3%)	0·04	6 (4%)

Data are mean number of events per patient or number of patients with at least one severe toxicity (%). An event was defined as the incidence of a toxicity assessed with the Common Toxicity Criteria for Adverse Events (CTCAE), version 4. Severe toxicity classified as grade 3, 4, or 5 on CTCAE, version 4. A toxicity that reached grade 3–5 in the acute phase and continued as grade 3–5 into the late phase was counted as both acute and late toxicities, but only counted once in the overall toxicity category. If a patient had two or more severe toxicities, they were still counted once in the total count.

There were significantly more serious adverse events with cisplatin than with cetuximab. 162 adverse events (mean rate of one event per patient) occurred in patients receiving cisplatin and 95 events (mean rate of 0·6 events per patient) occurred in patients receiving cetuximab (p<0·0001; appendix). The majority of serious adverse events (in 252 [98%] patients) resulted in admission to hospital. Serious adverse events in the cisplatin group were more likely (98 [61%] patients) to be assessed as related or possibly related to treatment than in the cetuximab group (18 [19%]; appendix). The most common serious adverse events for cisplatin were vomiting (in 48 [30%] patients) and nausea (in 46 [28%]), and those for cetuximab were vomiting (22 [13%]) and oral mucositis (21 [13%]; appendix). 114 (70%) serious adverse events with cisplatin resolved without sequelae compared with 59 (62%) with cetuximab.

**Figure 2:**
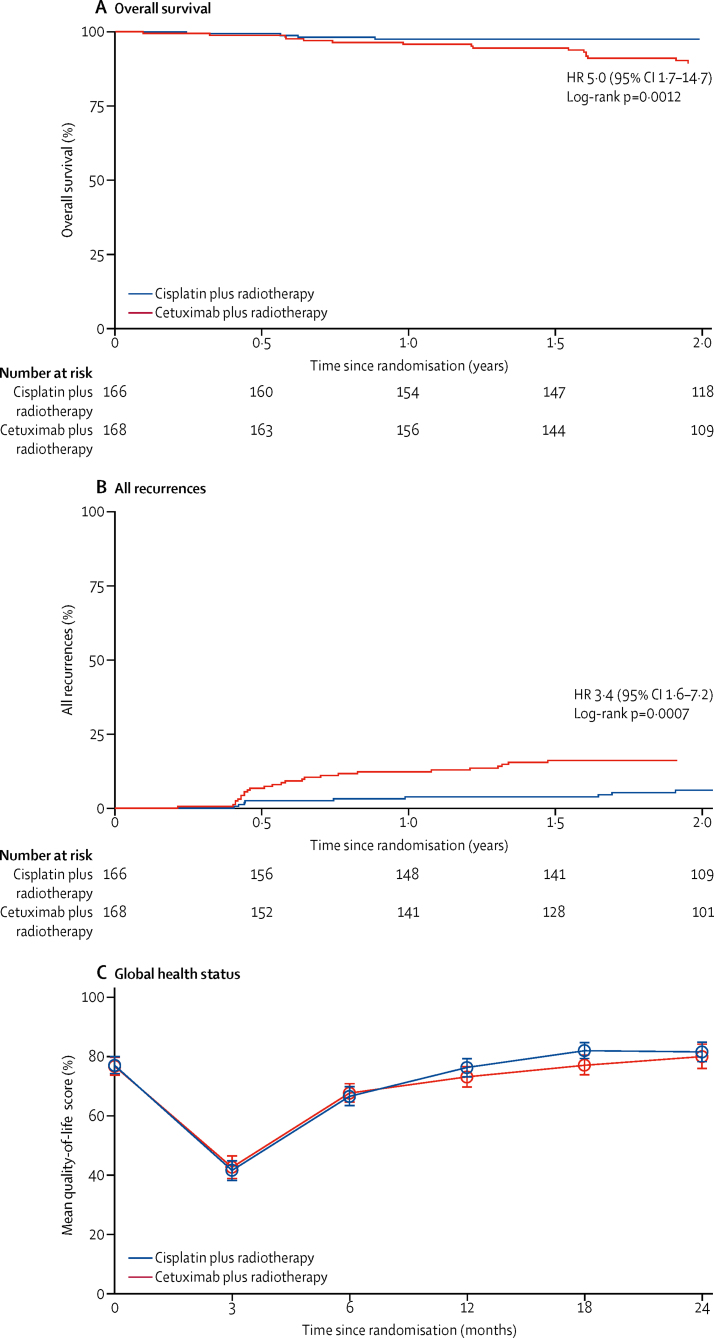
Overall survival, time to recurrence, and global quality of life scores, by treatment group (A) Kaplan-Meier estimates of overall survival, by treatment group. 2-year survival was 97·5% (95% CI 93·5–99·1) in the cisplatin group and 89·4% (83·2–93·4) in the cetuximab group (hazard ratio [HR] 5·0 [95% CI 1·7–14·7]; log-rank p=0·0012). (B) Time to any recurrence or distant metastasis, by treatment group. Persistent disease (occurring within 90 days of treatment completion) and new primaries are not included. The 1-year recurrence rate was 3·8% (95% CI 1·7–8·2) in the cisplatin group and 12·9% (8·6–19·1) in the cetuximab group. The 2-year recurrence rate was 6·0% (95% CI 3·2–11·3) in the cisplatin group and 16·1% (11·3–22·8) in the cetuximab group (HR 3·4 [95% CI 1·6 to 7·2]; log-rank p=0·0007). (C) Mean global quality-of-life score over time, by treatment group, measured by European Organisation for Research and Treatment of Cancer Quality-of-Life Questionnaire–Core 30 general (EORTC QLQ C30). Score 0 is worst quality of life and 100 is best quality of life; minimum clinically important difference 10 points (p=0·27).

A significant difference in 2-year overall survival was observed between cisplatin and cetuximab (97·5% *vs* 89·4%, HR 5·0 [95% CI 1·7–14·7], log-rank p=0·0012; figure 2A) and in the 2-year recurrence rate (6·0% *vs* 16·1%, 3·4 [1·6–7·2]; log-rank p=0·0007; figure 2B), in favour of cisplatin. After adjusting for known prognostic factors, the effect of treatment on overall survival (HR 5·9 [95% CI 2·0–17·8]; p=0·0015) and recurrence (3·9 [1·8–8·2]; p=0·0004) remained significant. Giving cetuximab instead of cisplatin was estimated to lead to one extra death at 2 years for every 12 patients treated (number needed to harm 12·3 [95% CI 7·0–50·8]). Similar results were seen for all above outcomes in the per-protocol population (appendix).

Overall, six patients died in the cisplatin group versus 20 in the cetuximab group. Cancer-related deaths occurred in four patients in the cisplatin group and in 15 in the cetuximab group. Four (2·5%) patients had persistent disease after cisplatin therapy, compared with five (3%) after cetuximab therapy. Significantly fewer recurrences were observed with cisplatin than with cetuximab (ten [6%] *vs* 29 [18%]; log-rank p=0·0007; appendix). Significantly fewer locoregional recurrences (3% *vs* 12%, log-rank p=0·0026) and fewer distant metastases (3% *vs* 9%, log-rank p=0·0092) were observed with cisplatin than with cetuximab. Five (3%) patients in each group developed second primaries. 11 (7%) patients in the cisplatin group had neck dissection for possible persistent disease at the 3-month post-treatment assessment point, and none for recurrent disease after this timepoint. Ten (6%) patients in the cetuximab group had neck dissection for possible persistent disease at the 3-month post-treatment point, and two for recurrent disease after this timepoint.

We did two post-hoc subgroup sensitivity analyses. In the 276 patients with TNM8 stage I or II disease, a significant difference in 2-year overall survival was observed: 98·4% (95% CI 93·9–99·6) for the cisplatin group (n=133) and 93·2% (87·4–96·4) for the cetuximab group (n=143; HR 4·3 [95% CI 0·9–19·8], log rank p=0·0431; appendix). In particular, the 58 patients with TNM8 stage III (T4 or N3) disease showed a larger 2-year overall survival detriment with cetuximab (67·1% [95% CI 42·5–83·1]) than with cisplatin (93·3% [75·9–98·3], HR 4·8 [95% CI 1·0–23·3], log rank p=0·0304; appendix).

In the 304 (94%) patients who tested doubly positive for p16 and HPV-DNA, there was still a significant difference in 2-year overall survival between cisplatin (97·2% [95% CI 92·8–99]) and cetuximab (89·7% [83·2–93·8]; HR 4·4 [95% CI 1·5–13·1]; log rank p=0·0035; appendix).

The mean global quality-of-life score on EORTC QLQ-C30 did not differ significantly between treatment groups at any of the timepoints (mean difference at 24 months of 1·51 points in favour of cisplatin, p=0·9976; figure 2C). A significant difference in social functioning was observed in favour of cetuximab at the end of treatment (mean difference of 8·67 points, p=0·0374), but this difference disappeared 6 months later. At 12 months and 24 months, a significant difference in role functioning was observed in favour of cisplatin (difference in mean scores of 8·32 points, p=0·0173; appendix). None of the differences reached the minimal clinically important difference of 10 points.

In terms of swallowing, no significant differences were observed between the treatment groups as assessed by the global M.D. Anderson Dysphagia Inventory score (mean difference at 24 months of 6·90 points in favour of cisplatin, p=0·1279; appendix). The only significant differences between the groups in the domains occurred at 12 months after treatment, and all were in favour of cisplatin (mean difference in emotional domain of 5·13 points, p=0·0151; functional domain difference of 3·96 points, p=0·0319; physical difference of 6·40 points, p=0·0113; and overall function difference of 5·49 points, p=0·0073), but none reached the minimal clinically important difference of 10 points, and all differences became non-significant again by 24 months.

## Discussion

HPV-positive oropharyngeal squamous cell carcinoma is projected to become the most common form of head and neck cancer in many developed countries.^21^, ^22^ Results of our study show that, in the setting of low-risk oropharyngeal squamous cell carcinoma, the use of cetuximab bioradiotherapy instead of cisplatin-based chemoradiotherapy resulted in no overall benefit in terms of toxicity but showed significant detriment in tumour control. Our trial also highlights that the good survival outcomes of HPV-positive low-risk oropharyngeal squamous cell carcinoma are in part a function of the type of treatment received, and not merely a reflection of favourable intrinsic tumour biology. Therefore, cisplatin-based chemoradiotherapy should continue to be considered the standard of care in this setting.

Contrary to expectations from the findings of the initial cetuximab registration trial,^11^ the rates of early, late, and overall serious toxicity resulting from cetuximab were not lower than those of cisplatin, although the toxicity profile was different. Global quality of life also appeared to be similar. Less than half of patients received the full dose of cisplatin because of toxicity. However, there was still a significant difference in survival compared with the cetuximab group in which most patients received the full regimen. Although cetuximab does result in fewer serious adverse events, this alone is not sufficient justification for its use in this setting. The findings of our study confirm those of other studies and meta-analyses comparing chemotherapy to bioradiotherapy with EGFR inhibitors in mixed head and neck cancer populations.^23^, ^24^ Until now, to our knowledge, no randomised studies had been done in HPV-positive disease.

As HPV-positive oropharyngeal squamous cell carcinoma is a relatively new disease entity, there have been changes in risk classification schemes, its TNM staging system, and the use of p16 immunohistochemistry as the sole determinant of HPV positivity since the inception of this trial and during its conduct. We defined low-risk patients according to the Ang classification,^4^ which is the most widely cited and used system. Since then, many other prognostic systems have been developed for this indication, each with different component factors. The new AJCC/UICC TNM 8th edition has included a new classification for HPV-positive oropharyngeal squamous cell carcinoma. In this new classification, HPV-positive T4 and N3 cases, constituting stage III HPV-positive oropharyngeal squamous cell carcinoma, have been shown to have higher rates of distant metastases than stage I or II tumours.^25^ Additionally, the use of p16 alone as a surrogate marker of HPV causation has been criticised, because in some studies the subsets comprising p16-positive, HPV-DNA-negative disease appear to have poorer outcomes, similar to patients with p16-negative and HPV-DNA-negative disease.^26^ In other studies, this subset of patients showed similar survival to patients with p16-positive, HPV-positive disease.^27^ To understand whether these factors affected our conclusions, we did post-hoc sensitivity analyses and found no difference in the results. Patients with T4 or N3 disease appeared to have even higher detriments in overall survival if they were treated with cetuximab than if they were treated with cisplatin. The numbers were small, however, leading to wide confidence intervals.

Our study was originally designed to test for differences in toxicity as a means of assessing whether cetuximab effectively reduces treatment-related toxicity for patients with HPV-positive oropharyngeal squamous cell carcinoma. We anticipated equivalent disease control and survival endpoints between study groups and did not formally power the study to show non-inferiority. Despite this limitation, the trial shows significant differences in both recurrence and survival endpoints in favour of cisplatin-based chemoradiotherapy. This outcome appears to be due to a relatively larger effect of cisplatin, compared with cetuximab, on locoregional control and distant metastases. We used p16 and HPV DNA in-situ hybridisation to assess HPV status, as this method is widely accepted^16^ and is recommended by the US National Comprehensive Care guidelines as one of the appropriate options for this purpose.^28^ This could be viewed as a limitation, because HPVE6/E7 RNA evaluation by PCR is considered the gold standard for testing HPV status. However, this method is not easily feasible in the clinical setting; moreover, in our study, patients who were p16-positive and HPV-positive showed similar survival rates to those who were p16-positive and HPV-negative.

Studies testing different de-escalation approaches for low-risk and intermediate-risk HPV-positive oropharyngeal squamous cell carcinoma are underway. Our data suggest that cisplatin-based chemoradiotherapy delivers substantially improved outcomes compared with bioradiotherapy with cetuximab, even in patients with good outcomes. Assuming that cetuximab does not reduce the efficacy of radiotherapy to control disease, these findings support the beneficial effects of adding cisplatin to radiotherapy in this group of patients. The reduced rate of distant metastasis also suggests that cisplatin might contribute to a systemic effect, even in low-risk patients. In light of our findings, we would advise caution with de-escalation strategies, especially those that remove systemic chemotherapy altogether, and strongly advocate that the results of phase 3 trials should be awaited before making any changes to routine clinical management.

This study is, to our knowledge, one of the first to compare cetuximab with cisplatin in combination with radiotherapy in the context of treatment de-escalation in HPV-positive disease. Not only did this trial show no reduction in toxicity with cetuximab but it also confirmed a statistically and clinically significant detriment in tumour control and survival endpoints with this therapy. These results have immediate implications for clinical practice and highlight the importance of doing comparative phase 3 trials in new indications, even for treatments that are already approved or have shown benefit in phase 2 trials.

## Data sharing
