# Supplementary material for: Radiotherapy plus cisplatin or cetuximab in low-risk human papillomavirus-positive oropharyngeal cancer (De-ESCALaTE HPV): an open-label randomised controlled phase 3 trial
Source: Lancet. 2019 Jan 5;393(10166):51–60. doi: 10.1016/S0140-6736(18)32752-1 (PMC6319250; doi:10.1016/S0140-6736(18)32752-1)
Supplement: Supplementary appendix [file mmc1.pdf]

THE LANCET

Supplementary appendix

This appendix formed part of the original submission and has been peer reviewed. We post it as supplied by the authors.

Supplement to: Mehanna H, Robinson M, Hartley A, et al. Radiotherapy plus cisplatin or cetuximab in low-risk human papillomavirus-positive oropharyngeal cancer (De-ESCALaTE HPV): an open-label randomised controlled phase 3 trial. *Lancet* 2018; published online Nov 15. http://dx.doi.org/10.1016/S0140-6736(18)32752-1.

Supplementary Appendix

Supplement to Mehanna H, Robinson M, Hartley A et al

Radiation with cisplatin or cetuximab in low-risk oropharyngeal cancer.

Contents

List of investigators	page	2
Supplementary information on quality assurance processes	page	6
Supplementary information on statistical analysis methods	page	10
Supplementary Tables S1-S7 and Figures S1 and S4	page	15
References and data sharing statement	page	26

8 Full list of De-ESCALaTE trial investigators

Site	PI
Aberdeen	Rafael Moleron
Beaumont Hospital	Orla McArdle
Bradford	Karen Dyker
Bristol	Hoda Al Booz
Castle Hill	Lorcan O'Toole
Cheltenham	Audrey Cook
Clatterbridge	David Husband
Colchester	Vivienne Loo
Glan Clwyd	Win Soe
James Cook Uni	Eleanor Aynsley
Leicester	Thiagarajan Sridhar

Musgrove Park	Petra Jankowska
New Cross	Mano Joseph
Norfolk and Norwich	Konstantinos Geropantas
Northampton	Deepali Vaidya
Nottingham	Matthew Griffin
QE Birmingham	Andrew Hartley
Royal Derby	Rengarajan Vijayan
Royal Devon & Exeter	David Hwang
Royal Marsden	Kevin Harrington
Royal Shrewsbury	Laura Pettit
Royal Surrey	Stephen Whitaker
Royal United Hospital	Emma De Winton
Singleton Hospital	Martin Rolles

SLRON @ St Luke's (Dublin)	Sinéad Brennan
St. James' Institute	Mehmet Sen
UCL	Ruheena Mendes<br>Martin Forster
UHCW	Andrew Chan
Velindre	Mererid Evans
VUMC (Netherlands)	Jan Buter
Western General	Devraj Srinivasan
Weston Park	Bernie Foran
University of Birmingham	Paul Nankivell<br>Neil Sharma<br>Jennifer Bryant<br>Rachel Spruce<br>Nikos Batis

	Jill Brooks
National Radiotherapy QA group	Tom Roques<br>Margaret Bidmead<br>Huiqi Yang<br>Christopher Nutting<br>Justine Tyler<br>Julia Henderson<br>Helen Baines<br>Anne Gasnier<br>Elizabeth Miles<br>Catharine Clark<br>Mererid Evans

Supplementary information on quality assurance process

All treating hospitals had to be approved as head and neck treatment centers by their country's health authorities. All centres and oncologists completed the trial's central radiotherapy quality assurance accreditation. All oncologists, surgeons, radiologists and pathologists participating in the study had to be core members of the approved multi-disciplinary team, fulfilling minimum national qualifications, case throughput and quality assurance criteria.

Radiotherapy quality assurance

Randomization was stratified by center to ensure that equal numbers of patients treated and planned by each oncologist and using the same radiotherapy regimens were randomized to the two treatment arms.

Centers had to receive radiotherapy trial quality assurance (RTTQA) credentialing for intensity-modulated radiotherapy (IMRT) in order to enter patients into the De-ESCALaTE trial. An overview of the complete credentialing process, along with the associated data and documentation, is given on the National RTTQA group website (www.rttrialsqa.org.uk).The IMRT credentialing programme consisted of five modules:

1) Outlining benchmark cases: Quality assurance of the performance of clinicians in the outlining process was assessed by sending CT scans of test patients to each center participating in the study for outlining. Adherence to the trial protocol for the outlining was assessed and analysed by an independent expert member of the central RTTQA team.

2) Planning benchmark cases: Quality assurance of the planning technique was monitored by sending a pre-outlined test patient to each center for planning. Adherence to the protocol and plan quality was assessed and analysed by an independent expert member of the central RTTQA team.

3) Processes document: was submitted by the center detailing all aspects of the tasks required for a complete IMRT pathway.

4) A baseline questionnaire and a trial-specific questionnaire containing questions on a range of aspects relevant to the trial were completed by the center.

5) Dosimetry audit site visit: consisted of an output measurement and dosimetric measurements of a treatment plan. Audits were carried out either by the RTTQA group in person.

Some of the processes were common to other H&N trials running in the UK and, therefore, centers that had previously been credentialed for other trials were streamlined.

Changes in technique

If centers changed their outlining or planning technique during the course of the trial, they were required to repeat the QA where appropriate.

Prospective Case Reviews

The outlining and treatment plans for the first three clinical cases entered into the trial (at least one lateralised and one non-lateralised) from each center had to be reviewed and approved by the central RTTQA team before treatment could commence.

Trial Data Collection

Data were collected by the quality assurance team for all patients treated in the De-ESCALaTE trial. This included: CT images, MRI scans (when applicable), contours, plan and plan dose cubes along with completed plan assessment forms and dose-volume histograms (DVHs). Data were appropriately anonymised.

Histopathological Quality Assurance

Quality assurance review of diagnostic samples was undertaken by the central pathology review team at Cellular Pathology, Royal Victoria Infirmary, Newcastle upon Tyne . p16 immunohistochemistry and high-risk HPV DNA in situ hybridization testing was undertaken for the UK and Ireland by the central laboratory - Cellular Pathology, Royal Victoria Infirmary, Newcastle upon Tyne, which is an NHS accredited clinical pathology laboratory. For the Netherlands, testing took place at the VU University Medical Center, Amsterdam. Neck dissection specimens were reported according to the standards and datasets for histopathology reporting of nodal excisions and neck dissection specimens associated with head and neck carcinoma.

Data quality monitoring

On receipt, all forms were checked for completeness and congruity. Forms containing empty data fields or data anomalies were queried with the site for resolution. Data was entered onto the trial database and any further anomalies were identified and queried with the site. Periodically, data underwent additional checks to ensure consistency between data submitted on CRFs.

Trial staff maintained regular communication with sites, through routine calls, mailings and/or meetings. In the event of persistent issues with the quality and/or quality of data submitted, an on-site monitoring visit was arranged. In such circumstances, patient notes and the investigator site files were reviewed during the visit. The representative from the De-ESCALaTE HPV Trial Office worked with the site staff to resolve issues, offered appropriate training if

necessary, and determined the site’s future participation in the trial.

An audit were arranged at a site if the Trial Management Group assessed that it is necessary. Audits were conducted by an independent team, determined by the Trial Management Group.

Supplementary information on statistical analysis

Monitoring

The IDSMC committee met in accordance with the De_ESCALaTE IDSMC charter. Their main objective was to advise the Trial Steering Committee as to whether there is evidence or reason why the trial should be amended or terminated based on recruitment rates, compliance, safety or efficacy. The IDSMC met after the first 50 patients have completed treatment and regularly thereafter. Confidential reports containing recruitment, protocol compliance, safety data and interim analyses of outcomes will be reviewed by the IDSMC.

Interim analyses of the primary outcome was presented to the IDSMC using conservative tests with significance determine by a p-value of 0.001 (to preserve the overall alpha level of 0.05). The first interim analysis was done when 200 patients have been recruited (i.e. at least 80 patients have completed treatment and reached 2 year follow-up). At this time the interim analyses of the secondary endpoints of acute and late toxicity werepresented as well as locoregional recurrence and overall survival.

Sample size assumptions:

Calais et al (1999) and Denis et al (2004) are the only studies to report early and late toxicity respectively on (the same cohort of) only oropharyngeal cancer patients receiving CRT. They report 202 acute severe grade 3-5 toxicities in 108 patients receiving CRT in the acute phase, and 18 late severe toxicities in 27 living patients. They also report that 56% of patients had a severe late toxicity (maximum grade method).

The sample size calculation assumes an average rate of 0.66 late severe toxicity events per patient, as per Denis et al (2004), and an acute toxicity event rate of 1.85 events per patient (Calais, 1999). Together these would constitute an average of 2.5 severe events per patient.

Recruiting 304 patients (152 in each arm) will allow reductions greater than 25% in overall number of severe (grade 3-5), acute and late toxicities to be detected with a 2-sided test at the 5% level of significance allowing for 10% drop out with greater than 90% power. If the event rate is lower than the estimated 2.5, this sample size also allows the detection of greater than 25% reductions in the overall severe toxicity events at an average rate of up to 1.9 events per patient with 85% power and 1.7 events per patient with 80% power.

Recruiting 304 patients also allows the detection of a 50% reduction in late severe toxicities with at least 90% power and a 25% or more reduction in acute severe toxicities with 85% power. The calculations were based on simulations and analysis by a Poisson model.

**Grade 3/4/5 toxicity events per patient**	**Power (25% reduction)**	**Power (33% reduction)**	**Power (50% reduction)**
0.5	32%	55%	94%
1.0	59%	84%	99%
1.5	77%	95%	99%
2.0	88%	99%	99%

2.5	93%	99%	99%
3.0	97%	99%	99%

The IDSMC on 12th December 2013 recommended that a Poisson distribution should not be assumed for the numbers of toxic events. The assumption of non-Poisson data and use of the Mann-Whitney U test may result in slightly lower or higher power, depending on the distribution.

Early data from the trial suggested that the number of acute serious toxic events per participant may be higher than the pre-trial estimate, e.g. >3 instead of 1.85. Simulations using control arm data similar to this suggested that the power will exceed that in the above text for all comparisons that include acute events.

Power estimates were revised on the basis of data accrued in June 2016. An increase of 10% in the number of patients randomised to 334 will allow the detection of a 25% reduction in all severe toxicities with 83% power and a 50% reduction in late severe toxicities with 77% power.

Definitions of toxicity

Severe toxic event: defined as a toxicity assessed as grade 3-5 by CTCAE V4.0. The type of event was characterised by the CTCAE V4.0 System Organ Class and Term.

Acute toxic event: defined as occurring during treatment or less than 90 days after the end of treatment.

Late toxic event: defined as occurring between 90 days and two years after end of treatment.

Details of counting events within a period of interest

Multiple occurrences of events of a single toxicity type within an analysis time period are counted as a single event. Events that were present both in before 90 days after treatment and remained after that period were counted as acute events and also as late events, but were not double-counted when analysing the overall number of acute and late events.

Toxicities reported as part of a serious adverse event notification but that were not reported as toxicity event were added to the counts of toxicity events.

The numbers of patients affected by each of these toxicity categories were reported and compared.

For TAME analysis, the counts were categorised by system organ class, so as to be as close as possible to the Trotti et al method[1]:

1. The mean number of serious acute events per patient for each of the treatment regimens during the defined acute risk interval were calculated. (This is the T of TAME in Trotti, 2007[1]).

2. The mean number of serious late events per patient for each of the treatment regimens during the defined late risk interval were calculated. (This is the A of TAME).

3. The cumulative incidence of death due to toxicity from study entry up to 30 days after the completion of cancer treatment were calculated (M).

4.E=End results, i.e. the other outcomes.

Statistical analyses were performed using SAS V9.3 software.

Quality of life questionnaires

Quality of life aspects have not been specifically measured in HPV+ oropharyngeal patients to date. Indeed, there are very limited quality of life data specifically for oropharyngeal cancer patients, as most studies have reported on patients with a heterogeneous group of head and neck cancers. Therefore, there is no pilot data to base power calculations on. This trial allowed this to be quantified. We have followed the EORTC recommendations for reporting and comparing QoL results using EORTC quality of life questionnaires (http://groups.eortc.be/qol/downloads/200203guidelines_qol.pdf). The main outcomes of principal interest were overall QoL and swallowing, however we also reported the other domains. We have reported the mean (and standard deviation) of the overall and domain-specific scores for both treatment arm groups at each time point (baseline, 3 months, 6 months, 12 and 24 months).

Supplementary tables and figures

Supplementary table S1A: Completion rates of patient reported outcome questionnaires at each time point.

**Timepoint**	**Cisplatin+RT**	**Cetuximab+RT**	**Total**
	**Proportion (%)**	**Proportion (%)**	**Proportion (%)**
EORTC QLQ-C30			
Baseline	155/162 (95.7%)	153/165 (92.7%)	308/327 (94.2%)
End of Treatment	124/162 (76.5%)	136/165 (82.4%)	260/327 (79.5%)
3 months	131/162 (80.9%)	130/165 (78.8%)	261/327 (79.8%)
6 months	129/162 (79.6%)	127/165 (77%)	256/327 (78.3%)
12 months	129/162 (79.6%)	128/165 (77.6%)	257/327 (78.6%)
24 months	97/162 (59.9%)	93/165 (56.4%)	190/327 (58.1%)
MDADI			
Baseline	155/162 (95.7%)	153/165 (92.7%)	308/327 (94.2%)
End of Treatment	124/162 (76.5%)	136/165 (82.4%)	260/327 (79.5%)
3 months	131/162 (80.9%)	130/165 (78.8%)	261/327 (79.8%)
6 months	129/162 (79.6%)	127/165 (77%)	256/327 (78.3%)
12 months	129/162 (79.6%)	129/165 (78.2%)	258/327 (78.9%)
24 months	101/162 (62.3%)	96/165 (58.2%)	197/327 (60.2%)

Supplementary table S1B: Co-morbidities of participants.

**Comorbidity (Multiple comorbidities per patient are counted)**	**Cisplatin (N=163) # (%)**	**Cetuximab (N=165) # (%)**	**Total* (N=338) # (%)**
**Angina**	1 (0.6)	1 (0.6)	2 (0.6)
**Chronic pulmonary disease**	1 (0.6)	5 (3.0)	6 (1.8)
**Congestive cardiac failure**	1 (0.6)	-	1 (0.3)
**Diabetes**	17 (10.4)	10 (6.1)	27 (8.2)
**Hypertension**	45 (27.6)	36 (21.8)	81 (24.7)
**MI**	1 (0.6)	2 (1.2)	3 (0.9)
**Stroke/TIA**	1 (0.6)	5 (3.0)	6 (1.8)
**Other reported comorbidities grouped**			
Asthma	8	4	12
Blood and lymphatic system disorders	-	1	1
Cardiac disorders	3	4	7
Ear and labyrinth disorders	1	-	1
Endocrine disorders	2	3	5
Eye disorders	1	1	2
Gastrointestinal disorders	3	7	10
Hepatobiliary disorders	1	1	2
Immune system disorders	1	2	3
Investigations (e.g. hypercholesterol, anaemia)	7	6	13
Metabolic and nutritional disorders	1	-	1
Musculoskeletal and connective tissue disorders	6	8	14
Neoplasms benign, malignant and unspecified	1	2	3
Nervous system	2	4	6
Psychiatric	3	8	11

Renal and urinary disorders	1	2	3
Reproductive system and breast disorders	2	2	4
Respiratory, thoracic and mediastinal disorders	1	1	2
Skin and subcutaneous tissue disorders	-	4	4
Vascular disorders	1	5	6
Other/Unclassifiable	6	13	19
**Total**	118	137	254
**Overall number of comorbidities reported per patient**	0.72	0.83	0.77

*= data not available on some patients

Supplementary table S1C: Baseline scores of Patient reported outcomes

	Treatment group					
	Arm A			Arm B		
	N	Mean	SD	N	Mean	SD
**QLQ C30 scores (general QoL)**	(Scale 0-100, high values are good)					
EORTC QLQ-C30 Global health status	153	77.2	17.2	150	76.6	18.3
EORTC QLQ-C30 Physical functioning	152	94.6	10.7	151	94.4	9.8
EORTC QLQ-C30 Role functioning	150	84.1	24.6	150	85.4	24.8
EORTC QLQ-C30 Emotional functioning	153	79.9	19.3	150	79.0	19.4
EORTC QLQ-C30 Cognitive functioning	153	90.7	15.3	149	89.4	18.0
EORTC QLQ-C30 Social functioning	153	83.0	23.4	149	82.8	20.9
**QLQ C30 Symptoms and side effects**	(Scale 0-100, low values are good)					
EORTC QLQ-C30 Fatigue	152	19.7	21.8	151	17.6	17.9
EORTC QLQ-C30 Nausea and vomiting	152	3.6	10.7	150	2.9	7.4
EORTC QLQ-C30 Overall pain	151	19.5	24.8	149	19.6	20.8
EORTC QLQ-C30 Dyspnoea	152	5.5	13.0	151	5.5	14.1
EORTC QLQ-C30 Insomnia	151	32.0	27.2	150	28.9	30.1
EORTC QLQ-C30 Appetite loss	152	13.4	22.5	151	16.3	25.8
EORTC QLQ-C30 Constipation	152	8.3	17.7	151	12.1	22.3
EORTC QLQ-C30 Diarrhoea	153	5.2	14.4	150	4.7	13.9
EORTC QLQ-C30 Financial difficulties	153	19.4	29.5	150	20.0	30.4
**HN35 scores (Head & neck QoL)**	(Scale 0-100, high values are good)					
EORTC HN35 Pain in head and neck	153	76.1	26.1	151	74.5	22.7
EORTC HN35 Problems with swallowing	153	90.5	18.1	151	90.9	16.1
EORTC HN35 Problems with smell/taste	152	94.5	13.5	151	93.4	14.7
EORTC HN35 Problems with speech	153	91.1	16.8	152	91.7	12.8
EORTC HN35 Trouble with social eating	153	88.1	22.1	152	88.2	20.8
EORTC HN35 Trouble with social contact	153	95.5	12.7	152	96.0	9.0
EORTC HN35 Less sexuality	140	80.0	28.6	142	82.9	26.9
EORTC HN35 Problems with teeth	153	86.7	27.4	151	83.0	28.8

EORTC HN35 Problems opening mouth wide	153	90.0	22.3	151	88.1	24.1
EORTC HN35 Dry mouth	153	83.4	27.6	151	84.5	24.6
EORTC HN35 Sticky saliva	153	89.3	21.5	151	90.3	17.5
EORTC HN35 Coughing	153	83.9	22.0	151	83.4	20.3
EORTC HN35 Felt ill	153	87.6	22.6	150	88.4	19.3
EORTC HN35 Used pain killers	152	41.4	49.4	152	31.6	46.6
EORTC HN35 Used nutritional supplements	153	82.4	38.2	152	83.6	37.2
EORTC HN35 Used a feeding tube	151	96.0	19.6	152	98.7	11.4
EORTC HN35 Weight loss - lower score means lost wt	153	71.2	45.4	152	75.7	43.1
EORTC HN35 Weight gain - lower score means gained wt	152	82.9	37.8	152	83.6	37.2
**MDADI (Dysphagia scale)**	(Scale 0-100, high values are good)					
MDADI dysphagia global	145	81.0	28.0	145	85.5	23.7
MDADI dysphagia emotional	149	79.8	18.3	148	83.7	15.1
MDADI dysphagia functional	149	82.9	18.9	148	84.7	16.1
MDADI dysphagia physical	149	80.9	23.9	147	83.7	21.0
MDADI dysphagia composite (overall function)	149	81.1	19.8	148	83.9	16.3

Supplementary table S2: Numbers of cisplatin doses, and alternative carboplatin doses, received by patients

**Number of cycles of cisplatin**	**Number of doses of carboplatin**			**Total**
**Received by each patient**	No doses	1 dose	2 doses	
0	1 (0.7%)	-	-	1 (0.6%)
1	7 (4.7%)	4 (50%)	5 (100%)	16 (9.9%)
2	79 (53%)	4 (50%)	-	83 (51.2%)
3	62 (41.6%)	-	-	62 (38.3%)
Total	149	8	5	162

* 4 patients withdrawn before receiving treatment

Supplementary table S3: Numbers of cetuximab doses received by patients

Number of doses received	Number of participants (%)
8	130 (79.4%)
7	23 (13.9%)
6	0 (0%)
5	3 (1.8%)
4	2 (1.2%)
3	3 (1.8%)
2	3 (1.8%)
Total	164 (100%)*

*4 patients were withdrawn before receiving treatment

Supplementary table S4: Dose of radiotherapy (Gy) delivered, by arm

Amount (x)	Cisplatin+RT N (%)	Cetuximab+RT N (%)	Total N (%)	P-value
65<x<70	8 (4.9%)	4 (2.5%)	12 (3.7%)	0.256*
70	145 (89.5%)	152 (92.7%)	297	0.314
70<x<75	8 (4.9%)	8 (4.9%)	16	0.980
>75	1 (0.6%)	0 (0%)	1	0.497*
Total	162	164	326	

*p-value calculated using Fishers exact test, remainder by chi squared test

Supplementary table S5A: Proportion of patients affected by severe and all grade acute, late and overall (combined) toxicities, by treatment group

**Toxicity grouping**	**Proportion of patients affected by one or more toxicity events**	
	**Cisplatin+RT**	**Cetuximab+RT**
Acute period		
Grade 3/4/5	142/162 (87.6%)	145/165 (87.9%)
All grades	162/162 (100%)	165/165 (100%)
Late period		
Grade 3/4/5	46/156 (29.5%)	36/161 (22.4%)
All grades	156/156 (100%)	161/161 (100%)
Acute and late period		
Grade 3/4/5	145/162 (89.5%)	146/165 (88.5%)

Supplementary Table 5B: Mean number of acute, late and overall toxicity events per patient, by Common Toxicity Criteria Adverse Events (CTCAE) version 4.0 grade method for each of the treatment arms, as per protocol analysis .

Toxicity grouping	**Mean number of events per patient**		
	**Cisplatin+RT**	**Cetuximab+RT**	**P value**
**Acute (T of TAME)**			
Grade 3/4/5 (95% CI)	4.40 (3.85 to 4.95)	4.40 (3.88 to 4.91)	0.984
All grades	19.96 (18.80 to 21.12)	20.47 (19.30 to 21.65)	0.541
**Late (A of TAME)**			
Grade 3/4/5	0.42 (0.30 to 0.55)	0.48 (0.29 to 0.66)	0.638
All grades	9.59 (8.67 to 10.48)	9.96 (9.11 to 10.82)	0.543
**Overall**			
Grade 3/4/5	4.79 (4.21 to 5.39)	4.86 (4.25 to 5.47)	0.891
All grades	29.28 (27.45 to 31.12)	30.27 (28.46 to 32.08)	0.452

Supplementary table 5C: Toxicity outcomes for patients that have had complete regimen of treatment (3 doses for cisplatin and full 8 doses of cetuximab)

Toxicity grouping	**Mean number of events per patient**		
	**Cisplatin+RT N=62**	**Cetuximab+RT N=130**	**P value**
**Acute (T of TAME)**			
Grade 3/4/5 (95% CI)	4.13 (3.31 to 4.95)	4.11 (3.54 to 4.69)	0.977
Grade 3/4 (95% CI)	4.13 (3.31 to 4.95)	4.11 (3.54 to 4.69)	0.977
All grades	19.16 (17.27 to 21.05)	19.75 (18.49 to 21.01)	0.604
**Late (A of TAME)**			
Grade 3/4/5	0.26 (0.08 to 0.44)	0.41 (0.20 to 0.63)	0.365
All grades	9.08 (7.62 to 10.54)	9.46 (8.54 to 10.38)	0.654
**Overall**			
Grade 3/4/5	4.39 (3.49 to 5.28)	4.53 (3.85 to 5.21)	0.812
All grades	28.06 (25.09 to 31.04)	29.05 (27.13 to 30.96)	0.573

Supplementary table S6: relatedness of serious adverse events

Chi-squared test to compare groups, SAE= serious adverse events

**Could this event have been caused by the trial medication?**	**Cisplatin+RT**	**Cetuximab+RT**	**Total**
Definitely related to trial medication	42 (25.9%)	4 (4.2%)	46 (17.9%)
Probably related to trial medication	56 (34.6%)	14 (14.7%)	70 (27.2%)
Possibly related to trial medication	37 (22.8%)	34 (35.8%)	71 (27.6%)
Unlikely to be relate to trial medication	14 (8.6%)	21 (22.1%)	35 (13.6%)
Unrelated to trial medication	12 (7.4%)	22 (23.2%)	34 (13.2%)
Missing	1 (0.6%)	-	1 (0.4%)
Total	162	95	(P-value for trend <0.0001)

Supplementary Table S7: Causes of serious adverse events, by treatment group.

In alphabetical order of SAE event type

SAE Event	Cisplatin+RT		Cetuximab+RT		Total	
	Mean events	Proportion of patients (%)	Mean events	Proportion of patients (%)	Mean events	Proportion of patients (%)
Gastrointestinal disorders | Abdominal pain	0.02	2/162 (1.2%)	0.01	1/165 (0.6%)	0.01	3/327 (0.9%)
Renal and urinary disorders | Acute kidney injury	0.14	16/162 (9.9%)	-	-	0.07	16/327 (4.9%)
Gastrointestinal disorders | Anal ulcer	0.01	1/162 (0.6%)	-	-	0.00	1/327 (0.3%)
Blood and lymphatic system disorders | Anemia	0.02	3/162 (1.9%)	-	-	0.01	3/327 (0.9%)
Metabolism and nutrition disorders | Anorexia	0.15	16/162 (9.9%)	0.09	13/165 (7.9%)	0.12	29/327 (8.9%)
Respiratory, thoracic and mediastinal disorders | Aspiration	0.01	1/162 (0.6%)	0.02	3/165 (1.8%)	0.01	4/327 (1.2%)
Respiratory, thoracic and mediastinal disorders | Atelectasis	0.01	1/162 (0.6%)	-	-	0.00	1/327 (0.3%)
Cardiac disorders | Atrial fibrillation	-	-	0.01	1/165 (0.6%)	0.00	1/327 (0.3%)
Blood and lymphatic system disorders | Blood and lymphatic system disorders - Other, specify	0.02	4/162 (2.5%)	-	-	0.01	4/327 (1.2%)
Cardiac disorders | Cardiac disorders - Other, specify	-	-	0.01	1/165 (0.6%)	0.00	1/327 (0.3%)
Infections and infestations | Catheter related infection	-	-	0.01	1/165 (0.6%)	0.00	1/327 (0.3%)
Gastrointestinal disorders | Colitis	-	-	0.01	1/165 (0.6%)	0.00	1/327 (0.3%)
Gastrointestinal disorders | Colonic perforation	-	-	0.01	1/165 (0.6%)	0.00	1/327 (0.3%)
Psychiatric disorders | Confusion	0.01	1/162 (0.6%)	-	-	0.00	1/327 (0.3%)
Gastrointestinal disorders | Constipation	0.12	16/162 (9.9%)	0.04	6/165 (3.6%)	0.08	22/327 (6.7%)
Respiratory, thoracic and mediastinal disorders | Cough	0.01	2/162 (1.2%)	0.01	1/165 (0.6%)	0.01	3/327 (0.9%)
Investigations | Creatinine increased	0.02	4/162 (2.5%)	-	-	0.01	4/327 (1.2%)
Metabolism and nutrition disorders | Dehydration	0.21	26/162 (16%)	0.13	16/165 (9.7%)	0.17	42/327 (12.8%)

Injury, poisoning and procedural complications | Dermatitis radiation	-	-	0.04	6/165 (3.6%)	0.02	6/327 (1.8%)
Infections and infestations | Device related infection	0.01	1/162 (0.6%)	0.02	3/165 (1.8%)	0.01	4/327 (1.2%)
Gastrointestinal disorders | Diarrhea	0.04	7/162 (4.3%)	0.02	2/165 (1.2%)	0.03	9/327 (2.8%)
Gastrointestinal disorders | Dry mouth	-	-	0.01	1/165 (0.6%)	0.01	1/327 (0.3%)
Gastrointestinal disorders | Dyspepsia	0.02	2/162 (1.2%)	-	-	0.01	2/327 (0.6%)
Gastrointestinal disorders | Dysphagia	0.10	13/162 (8%)	0.08	10/165 (6.1%)	0.09	23/327 (7%)
Respiratory, thoracic and mediastinal disorders | Dyspnea	0.02	4/162 (2.5%)	0.01	1/165 (0.6%)	0.02	5/327 (1.5%)
General disorders and administration site conditions | Edema limbs	0.01	1/162 (0.6%)	-	-	0.00	1/327 (0.3%)
Gastrointestinal disorders | Esophageal pain	0.01	2/162 (1.2%)	-	-	0.01	2/327 (0.6%)
General disorders and administration site conditions | Fatigue	0.02	3/162 (1.9%)	0.02	3/165 (1.8%)	0.02	6/327 (1.8%)
Blood and lymphatic system disorders | Febrile neutropenia	0.04	6/162 (3.7%)	-	-	0.02	6/327 (1.8%)
General disorders and administration site conditions | Fever	0.08	12/162 (7.4%)	0.04	6/165 (3.6%)	0.06	18/327 (5.5%)
Musculoskeletal and connective tissue disorders | Flank pain	0.01	1/162 (0.6%)	-	-	0.00	1/327 (0.3%)
General disorders and administration site conditions | Flu like symptoms	0.01	1/162 (0.6%)	-	-	0.00	1/327 (0.3%)
Gastrointestinal disorders | Gastric hemorrhage	-	-	0.01	1/165 (0.6%)	0.00	1/327 (0.3%)
Gastrointestinal disorders | Gastroesophageal reflux disease	-	-	0.01	1/165 (0.6%)	0.00	1/327 (0.3%)
Gastrointestinal disorders | Gastrointestinal disorders - Other, specify	0.01	1/162 (0.6%)	0.02	4/165 (2.4%)	0.02	5/327 (1.5%)
Gastrointestinal disorders | Gastrointestinal pain	0.01	1/162 (0.6%)	-	-	0.00	1/327 (0.3%)
Respiratory, thoracic and mediastinal disorders | Hoarseness	-	-	0.01	1/165 (0.6%)	0.00	1/327 (0.3%)
Metabolism and nutrition disorders | Hypercalcemia	0.01	1/162 (0.6%)	0.01	1/165 (0.6%)	0.01	2/327 (0.6%)

Metabolism and nutrition disorders | Hyperkalemia	0.01	2/162 (1.2%)	0.01	1/165 (0.6%)	0.01	3/327 (0.9%)
Metabolism and nutrition disorders | Hypernatremia	0.01	1/162 (0.6%)	0.01	1/165 (0.6%)	0.01	2/327 (0.6%)
Metabolism and nutrition disorders | Hypokalemia	0.04	5/162 (3.1%)	0.02	3/165 (1.8%)	0.03	8/327 (2.4%)
Metabolism and nutrition disorders | Hypomagnesemia	0.01	1/162 (0.6%)	-	-	0.00	1/327 (0.3%)
Metabolism and nutrition disorders | Hyponatremia	0.01	1/162 (0.6%)	-	-	0.01	1/327 (0.3%)
Metabolism and nutrition disorders | Hypophosphatemia	0.01	1/162 (0.6%)	-	-	0.00	1/327 (0.3%)
Vascular disorders | Hypotension	-	-	0.02	4/165 (2.4%)	0.01	4/327 (1.2%)
Respiratory, thoracic and mediastinal disorders | Hypoxia	0.01	1/162 (0.6%)	-	-	0.00	1/327 (0.3%)
Infections and infestations | Infections and infestations - Other, specify	0.05	7/162 (4.3%)	0.02	4/165 (2.4%)	0.04	11/327 (3.4%)
Injury, poisoning and procedural complications | Injury, poisoning and procedural complications - Other, specify	-	-	0.01	1/165 (0.6%)	0.00	1/327 (0.3%)
Investigations | Investigations - Other, specify	0.03	4/162 (2.5%)	-	-	0.02	4/327 (1.2%)
Respiratory, thoracic and mediastinal disorders | Laryngeal mucositis	0.01	1/162 (0.6%)	-	-	0.00	1/327 (0.3%)
Cardiac disorders | Left ventricular systolic dysfunction	0.01	2/162 (1.2%)	-	-	0.01	2/327 (0.6%)
Nervous system disorders | Lethargy	0.01	1/162 (0.6%)	-	-	0.00	1/327 (0.3%)
Infections and infestations | Lung infection	0.01	1/162 (0.6%)	0.02	2/165 (1.2%)	0.02	3/327 (0.9%)
Metabolism and nutrition disorders | Metabolism and nutrition disorders - Other, specify	0.02	1/162 (0.6%)	0.01	1/165 (0.6%)	0.01	2/327 (0.6%)
Infections and infestations | Mucosal infection	0.01	1/162 (0.6%)	0.01	1/165 (0.6%)	0.01	2/327 (0.6%)
Gastrointestinal disorders | Mucositis oral	0.17	25/162 (15.4%)	0.17	21/165 (12.7%)	0.17	46/327 (14.1%)

Musculoskeletal and connective tissue disorders | Muscle weakness left-sided	-	-	0.01	1/165 (0.6%)	0.01	1/327 (0.3%)
Cardiac disorders | Myocardial infarction	0.01	1/162 (0.6%)	-	-	0.00	1/327 (0.3%)
Gastrointestinal disorders | Nausea	0.41	46/162 (28.4%)	0.12	16/165 (9.7%)	0.26	62/327 (19%)
Neoplasms benign, malignant and unspecified (incl cysts and polyps) | Neoplasms benign, malignant and unspecified (incl cysts and polyps) - Other, specify	-	-	0.01	1/165 (0.6%)	0.00	1/327 (0.3%)
Investigations | Neutrophil count decreased	0.02	3/162 (1.9%)	-	-	0.01	3/327 (0.9%)
General disorders and administration site conditions | Non-cardiac chest pain	0.01	1/162 (0.6%)	-	-	0.01	1/327 (0.3%)
Gastrointestinal disorders | Oral hemorrhage	0.01	2/162 (1.2%)	0.01	1/165 (0.6%)	0.01	3/327 (0.9%)
Gastrointestinal disorders | Oral pain	0.04	4/162 (2.5%)	0.07	6/165 (3.6%)	0.05	10/327 (3.1%)
General disorders and administration site conditions | Pain	-	-	0.01	1/165 (0.6%)	0.01	1/327 (0.3%)
Musculoskeletal and connective tissue disorders | Pain in extremity	0.01	1/162 (0.6%)	0.01	1/165 (0.6%)	0.01	2/327 (0.6%)
Nervous system disorders | Paresthesia	-	-	0.01	1/165 (0.6%)	0.00	1/327 (0.3%)
Cardiac disorders | Pericarditis	0.01	1/162 (0.6%)	-	-	0.00	1/327 (0.3%)
Vascular disorders | Peripheral ischemia	0.01	1/162 (0.6%)	-	-	0.00	1/327 (0.3%)
Respiratory, thoracic and mediastinal disorders | Pharyngeal hemorrhage	-	-	0.01	1/165 (0.6%)	0.00	1/327 (0.3%)
Respiratory, thoracic and mediastinal disorders | Pharyngeal mucositis	0.02	4/162 (2.5%)	0.02	3/165 (1.8%)	0.02	7/327 (2.1%)
Investigations | Platelet count decreased	0.01	1/162 (0.6%)	-	-	0.00	1/327 (0.3%)
Respiratory, thoracic and mediastinal disorders | Pneumonitis	0.01	1/162 (0.6%)	-	-	0.00	1/327 (0.3%)
Respiratory, thoracic and mediastinal disorders | Productive cough	0.01	1/162 (0.6%)	-	-	0.00	1/327 (0.3%)
Skin and subcutaneous tissue disorders | Pruritus	-	-	0.01	1/165 (0.6%)	0.00	1/327 (0.3%)
Skin and subcutaneous tissue disorders | Rash acneiform	-	-	0.01	1/165 (0.6%)	0.00	1/327 (0.3%)

Renal and urinary disorders | Renal and urinary disorders - Other, specify	0.02	2/162 (1.2%)	-	-	0.01	2/327 (0.6%)
Respiratory, thoracic and mediastinal disorders | Respiratory, thoracic and mediastinal disorders - Other, specify	0.01	1/162 (0.6%)	-	-	0.00	1/327 (0.3%)
Infections and infestations | Salivary gland infection	0.01	1/162 (0.6%)	-	-	0.00	1/327 (0.3%)
Infections and infestations | Sepsis	0.01	1/162 (0.6%)	-	-	0.00	1/327 (0.3%)
Investigations | Serum amylase increased	0.01	1/162 (0.6%)	-	-	0.00	1/327 (0.3%)
Cardiac disorders | Sinus tachycardia	0.02	2/162 (1.2%)	-	-	0.01	2/327 (0.6%)
Infections and infestations | Skin infection	0.01	1/162 (0.6%)	0.02	4/165 (2.4%)	0.02	5/327 (1.5%)
Skin and subcutaneous tissue disorders | Skin ulceration	-	-	0.01	1/165 (0.6%)	0.00	1/327 (0.3%)
Respiratory, thoracic and mediastinal disorders | Sore throat	0.04	5/162 (3.1%)	0.02	3/165 (1.8%)	0.03	8/327 (2.4%)
Infections and infestations | Stoma site infection	0.01	1/162 (0.6%)	-	-	0.00	1/327 (0.3%)
Gastrointestinal disorders | Stomach pain	0.02	1/162 (0.6%)	-	-	0.01	1/327 (0.3%)
General disorders and administration site conditions | Sudden death NOS	-	-	0.01	1/165 (0.6%)	0.00	1/327 (0.3%)
Nervous system disorders | Syncope	0.01	2/162 (1.2%)	0.01	2/165 (1.2%)	0.01	4/327 (1.2%)
Vascular disorders | Thromboembolic event	0.01	2/162 (1.2%)	0.01	1/165 (0.6%)	0.01	3/327 (0.9%)
Infections and infestations | Upper respiratory infection	0.01	2/162 (1.2%)	0.01	1/165 (0.6%)	0.01	3/327 (0.9%)
Renal and urinary disorders | Urinary retention	0.01	1/162 (0.6%)	-	-	0.00	1/327 (0.3%)
Infections and infestations | Urinary tract infection	0.01	2/162 (1.2%)	-	-	0.01	2/327 (0.6%)
Ear and labyrinth disorders | Vertigo	-	-	0.01	1/165 (0.6%)	0.00	1/327 (0.3%)
Ear and labyrinth disorders | Vestibular disorder	-	-	0.01	1/165 (0.6%)	0.00	1/327 (0.3%)
Gastrointestinal disorders | Vomiting	0.43	48/162 (29.6%)	0.17	22/165 (13.3%)	0.30	70/327 (21.4%)
Investigations | Weight loss	0.06	7/162 (4.3%)	0.03	5/165 (3%)	0.04	12/327 (3.7%)
Infections and infestations | Wound infection	0.01	2/162 (1.2%)	-	-	0.01	2/327 (0.6%)

Supplementary Figure S1A: Acute, late and overall (combined) toxicities, by severity and treatment group.

Severe toxicity classified as grade 3,4 or5, assessed using the Common Toxicity Criteria Adverse Events (CTCAE) version 4. All grades=CTCAE grades 0-5. A toxicity that reaches grade 3-5 in the acute phase and continues as Grade 3-5 into the late phase is counted as both an acute and a late severe toxicity, but only counted once in overall severe toxicity.

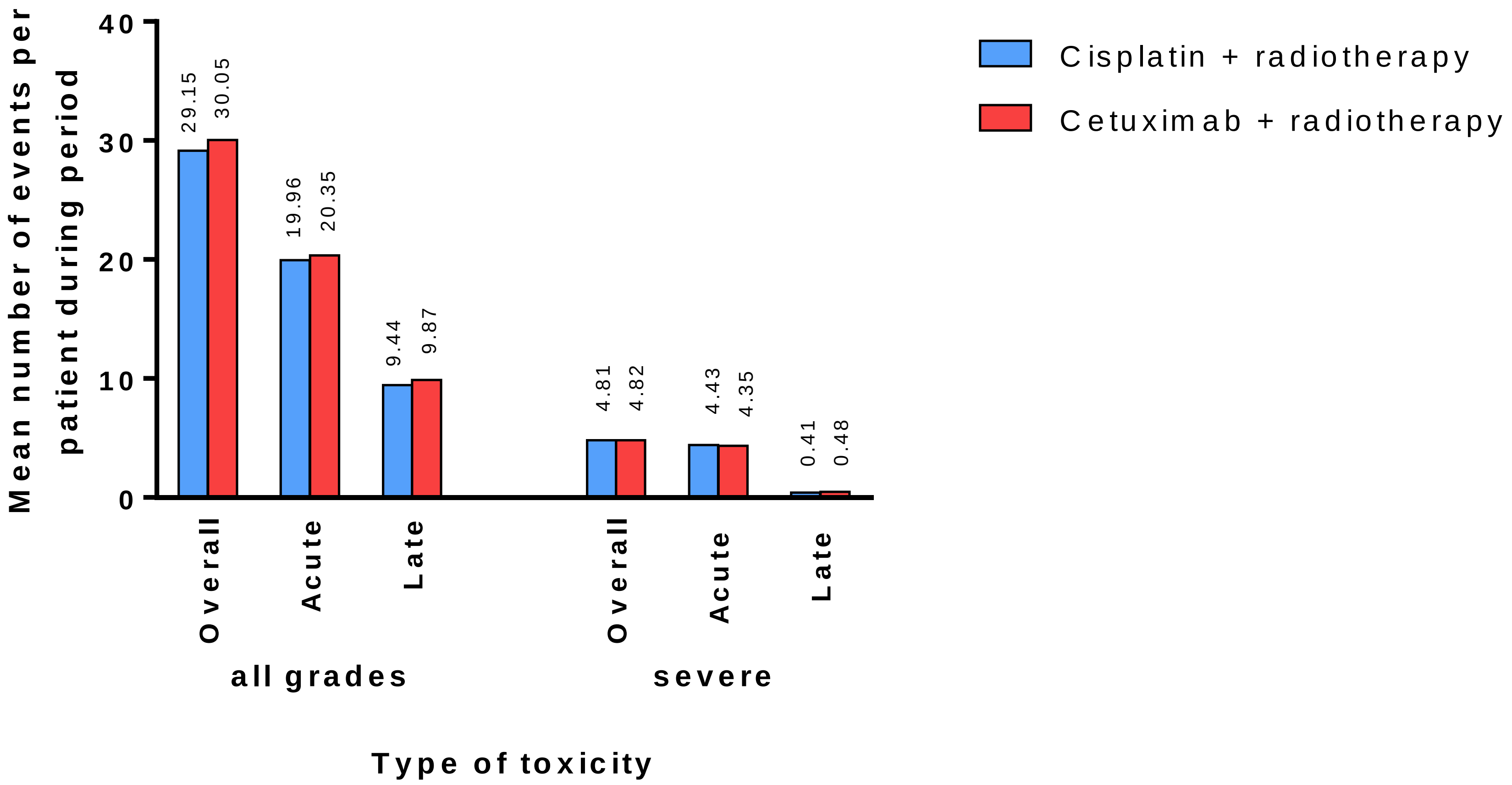

Supplementary Figure S1B: Serious adverse events by severity , by group

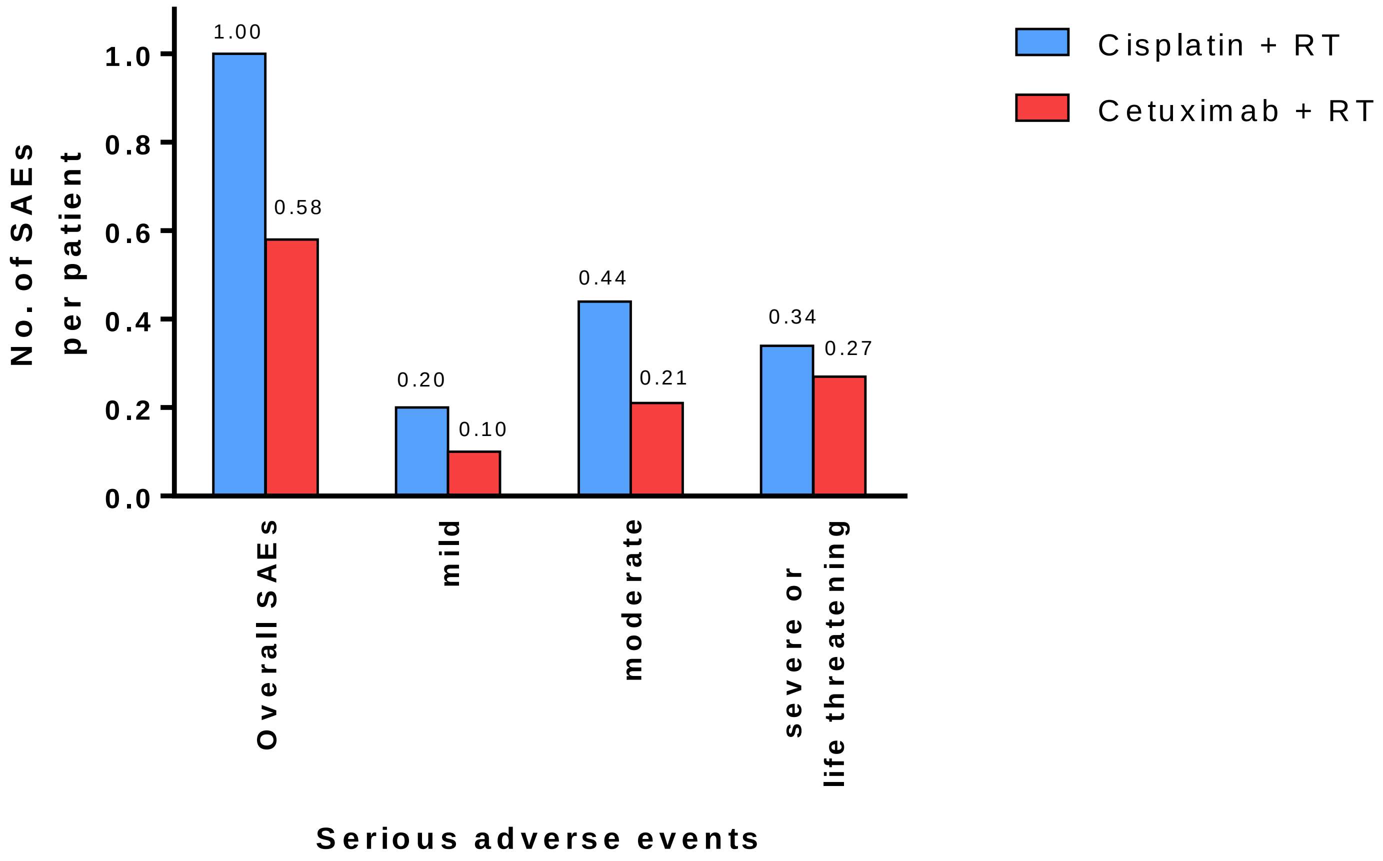

Supplementary Figure S2: Proportion of patients with persistent disease, loco-regional recurrence, distant metastases and second primaries, by treatment group. Some patients treated with cetuximab had both loco-regional recurrence and distant metastasis synchronously and are included in a separate grouping.

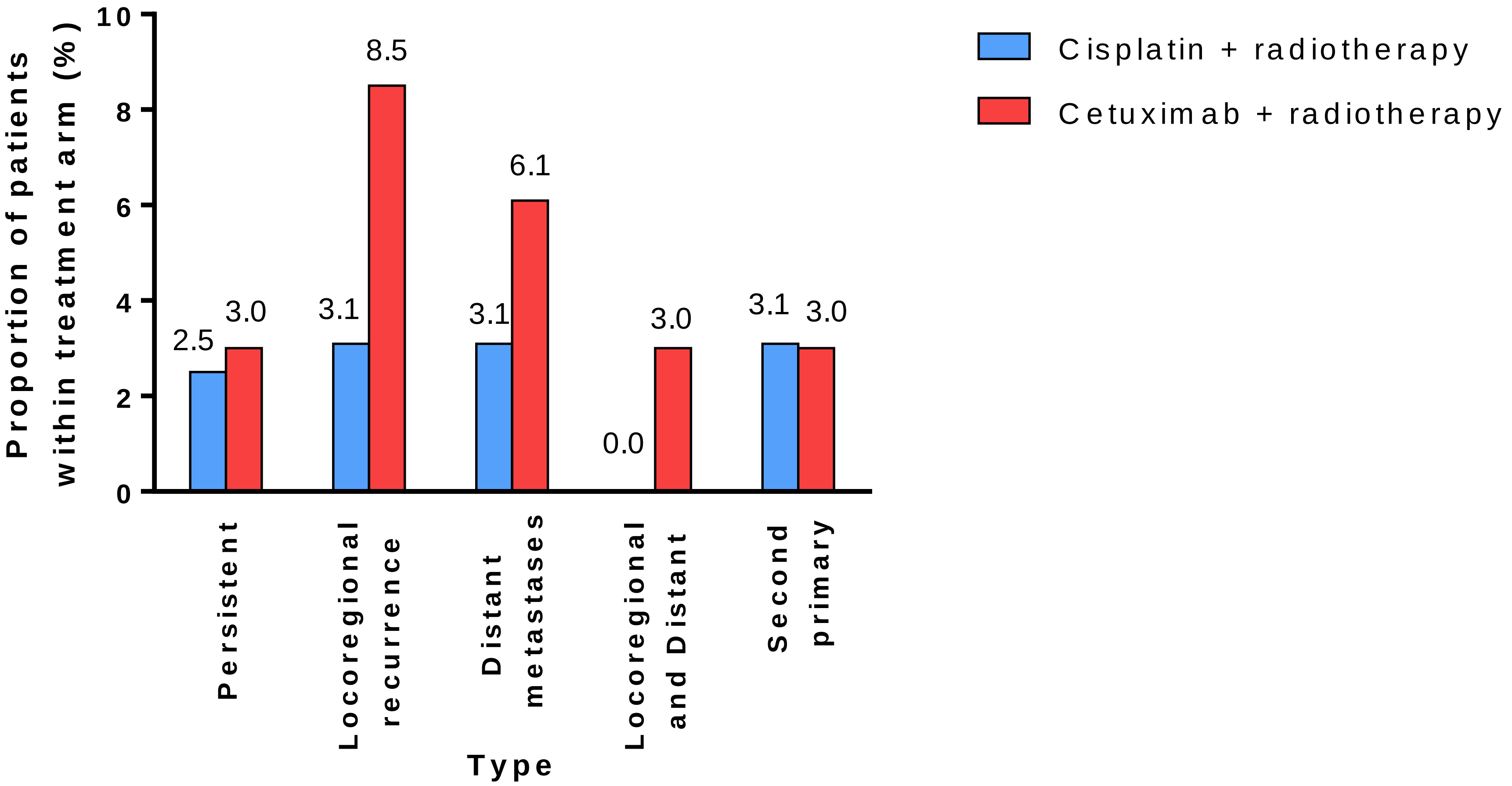

Supplementary Figure S3: Recurrences and post-hoc subgroup analyses

A. Overall survival of patients with TNM8 stage I and II disease (excludes patients with T4 and N3) n=276

The 2-year survival rate with 95% confidence interval is:

- 98.4% (93.9% to 99.6%) in the cisplatin arm
- 93.2% (87.4% to 96.4%) in the cetuximab arm

Hazards Ratio: 4.27, 95% CI: 0.92 to 19.75; Log rank p-value = 0.043

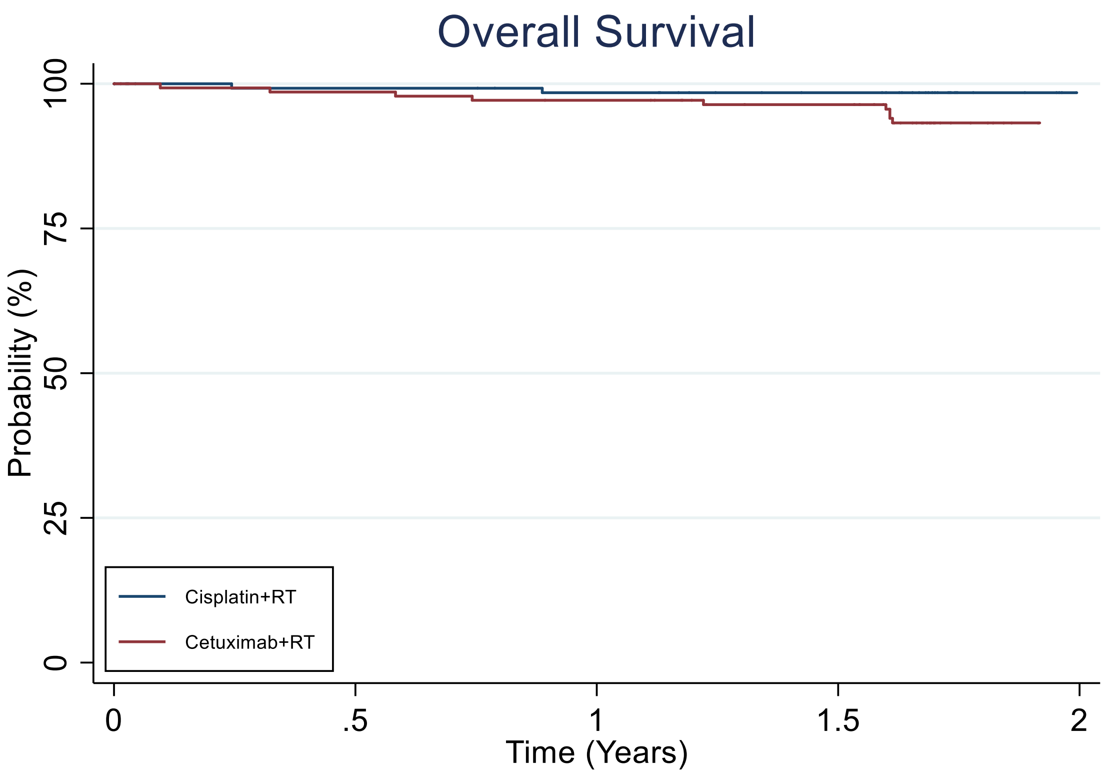

Number at risk					
Cisplatin+RT	133	129	126	119	95
Cetuximab+RT	143	138	135	127	97

B. **Overall survival for patients with T4 or N3 disease, by trial group. N=58.**

The 2-year survival rate with 95% confidence interval is 93.3% (75.9% to 98.3%) in the Cisplatin and Radiotherapy arm and 67.1% (42.5% to 83.1%) in the Cetuximab and Radiotherapy arm. Log-rank p-value= 0.03; HR: 4.83, 95% CI: 1.00 to 23.31

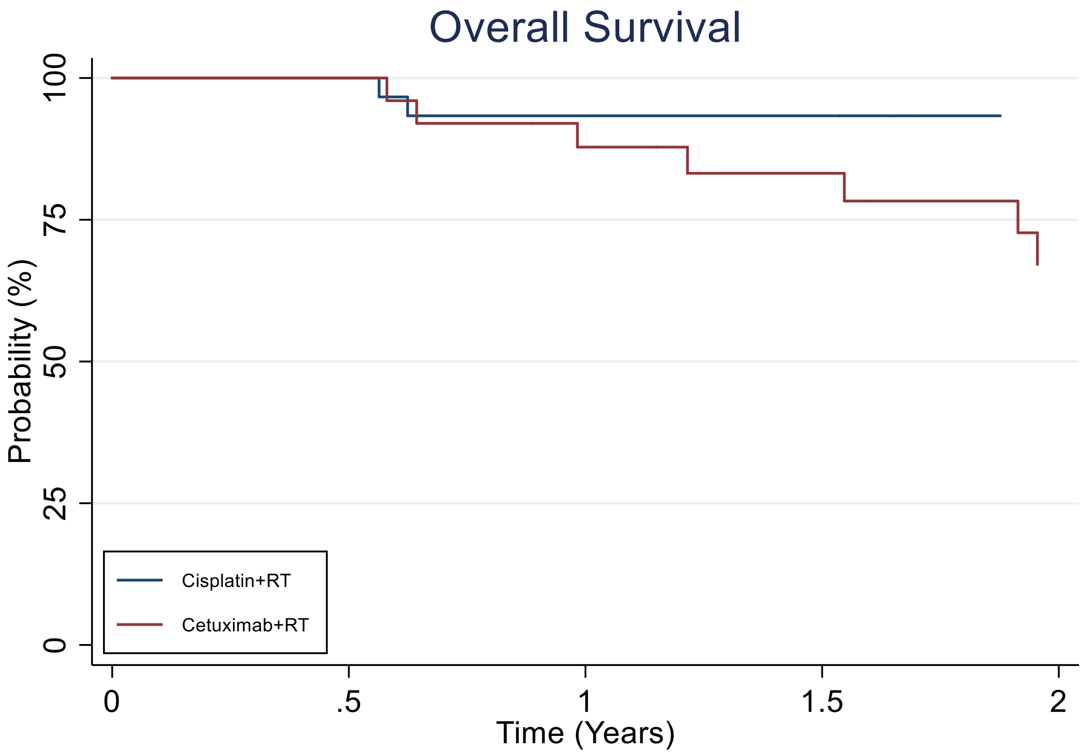

Number at risk					
Cisplatin+RT	33	31	28	28	23
Cetuximab+RT	25	25	21	17	12

C. Overall survival of patients with doubly-positive on p16 immunohostochemistry and High-Risk HPV DNA in-situ hybridization, n=304

The 2-year survival rate with 95% confidence interval is:

- 97.2% (92.8% to 99%) in the cisplatin arm
- 89.7% (83.2% to 93.8%) in the cetuximab arm Hazards Ratio 4.4, 95% CI: 1.49 to 13.11 Log rank p-value = 0.004

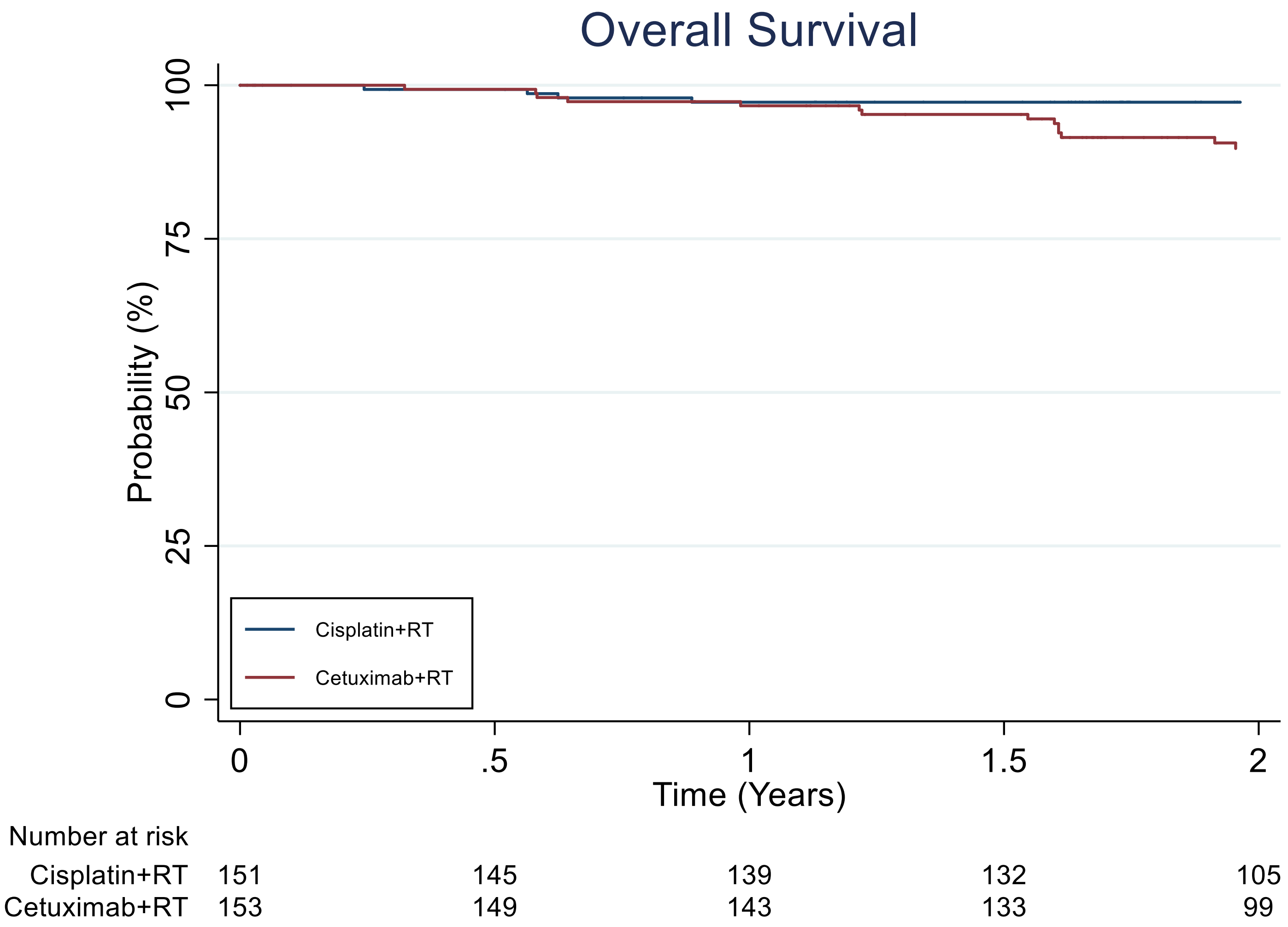

D. Kaplan-Meier curve for overall survival in per-protocol analysis .

2 year OS rate for Cisplatin: 97.5%, 95% CI: 93.4% to 99.1%; 2 year OS rate for Cetuximab: 90.0%, 95% CI: 83.8% to 93.8%, HR: 4.76, 95% CI: 1.61 to 14.05, log-rank p-value=0.0019

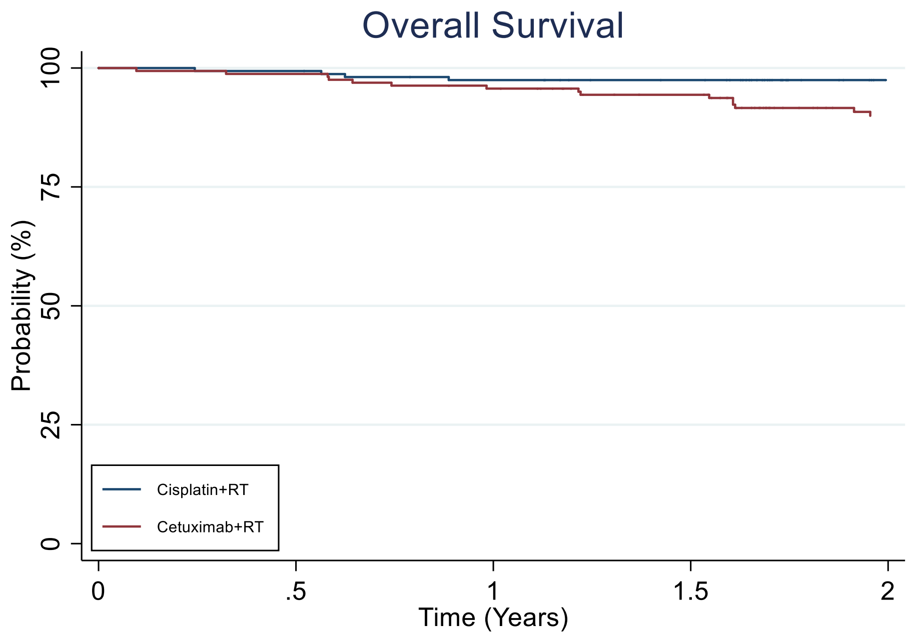

Number at risk					
Cisplatin+RT	159	158	153	147	118
Cetuximab+RT	162	160	154	142	108

Supplementary Figure S4: Quality of life measured by EORTC C30 questionnaire and swallowing measured by the MD Anderson Dysphagia Questionnaire (MDADI)

A. EORTC Physical functioning

P-value = 0.122

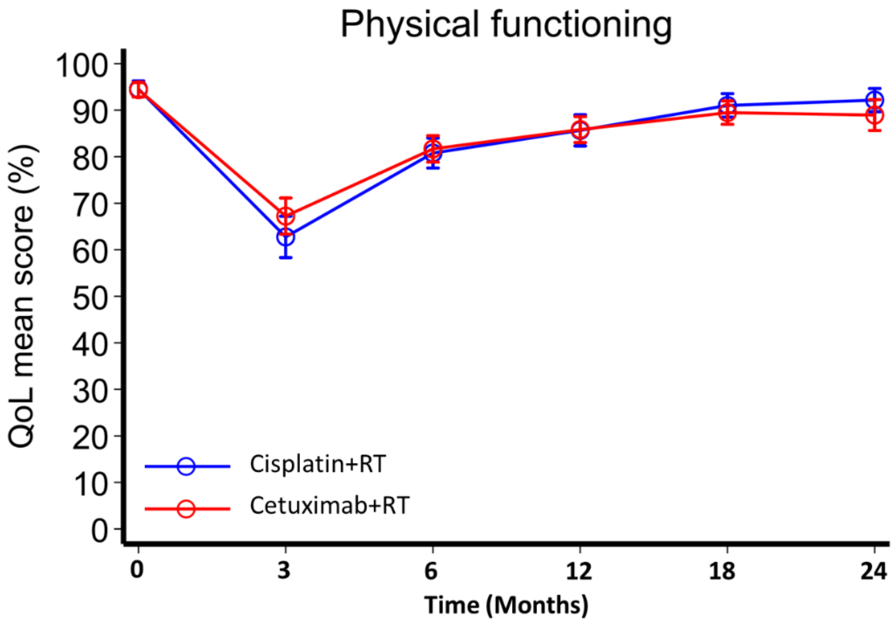

B. EORTC Role functioning

P-value = 0.121

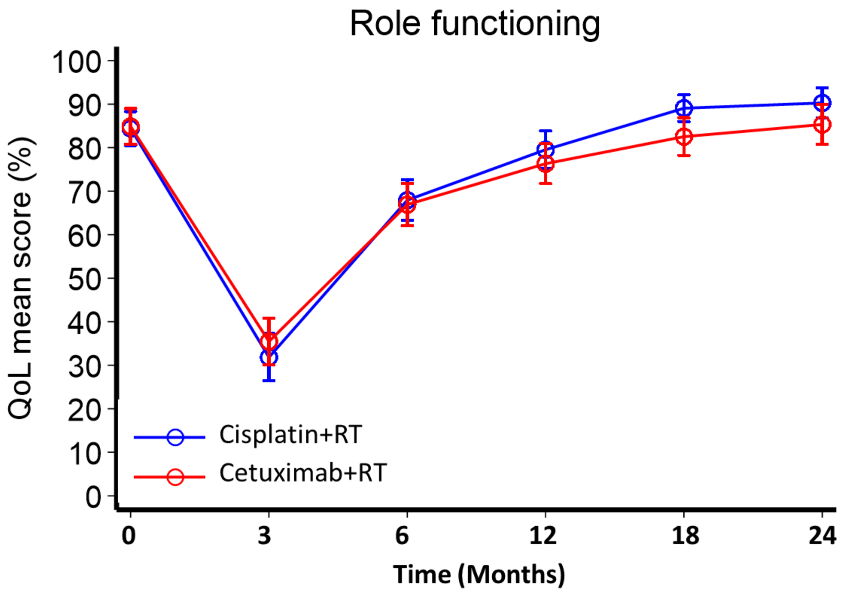

C. EORTC Emotional functioning

P-value = 0.851

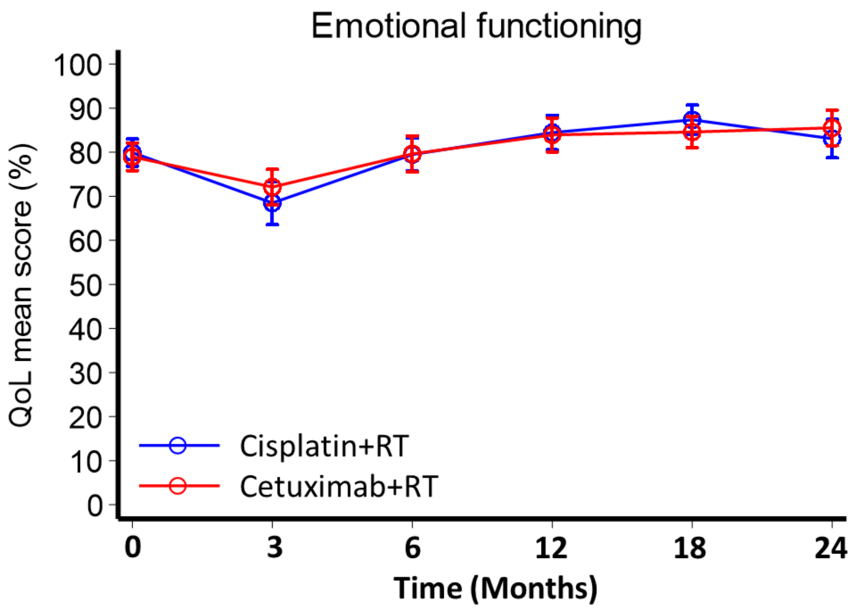

D. EORTC Cognitive functioning

P-value = 0.443

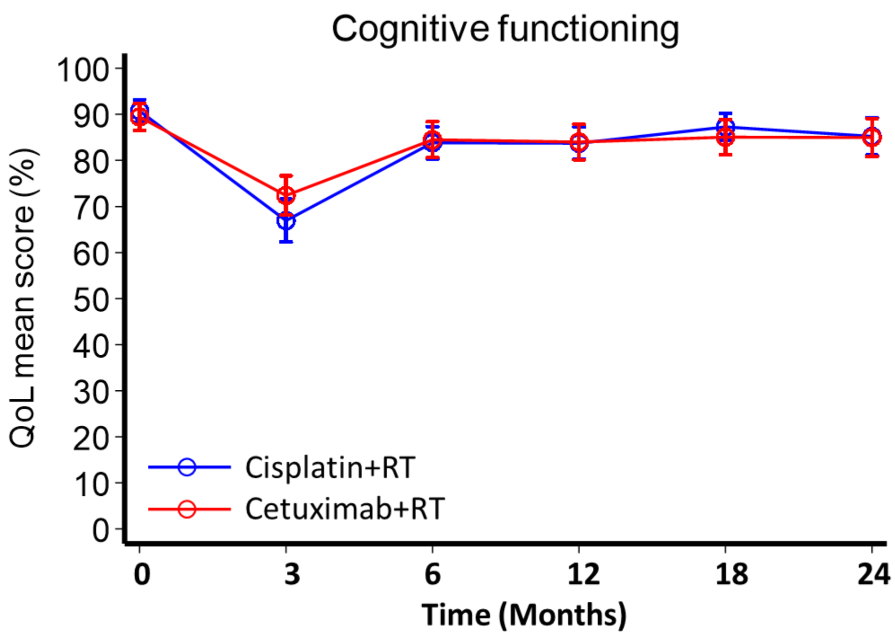

E. EORTC Social functioning

P-value = 0.182

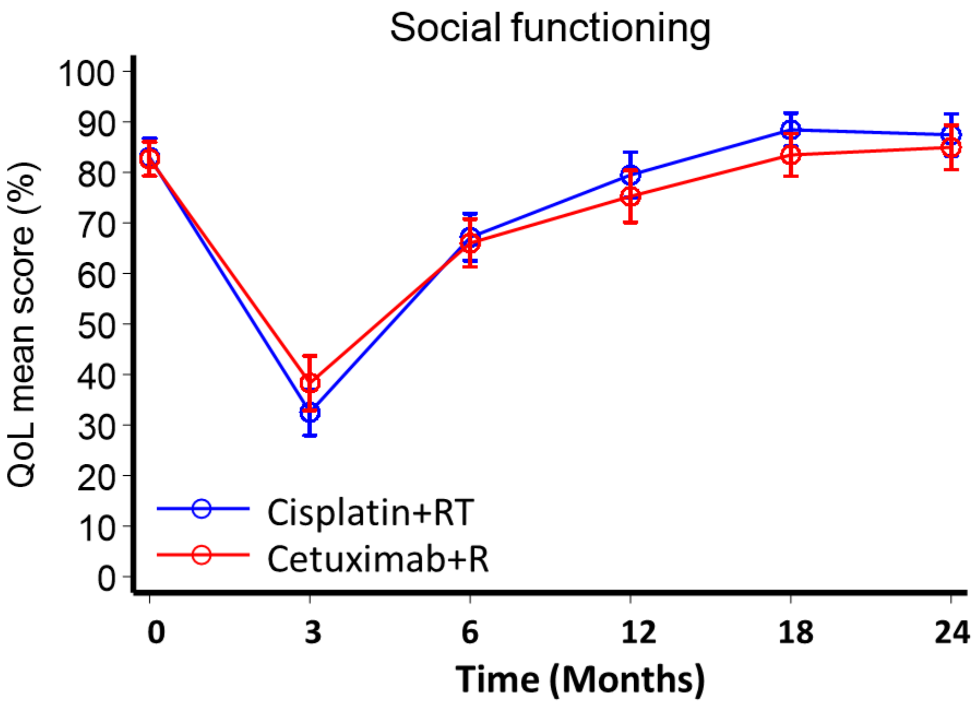

F. MDADI dysphagia global

P-value = 0.0948

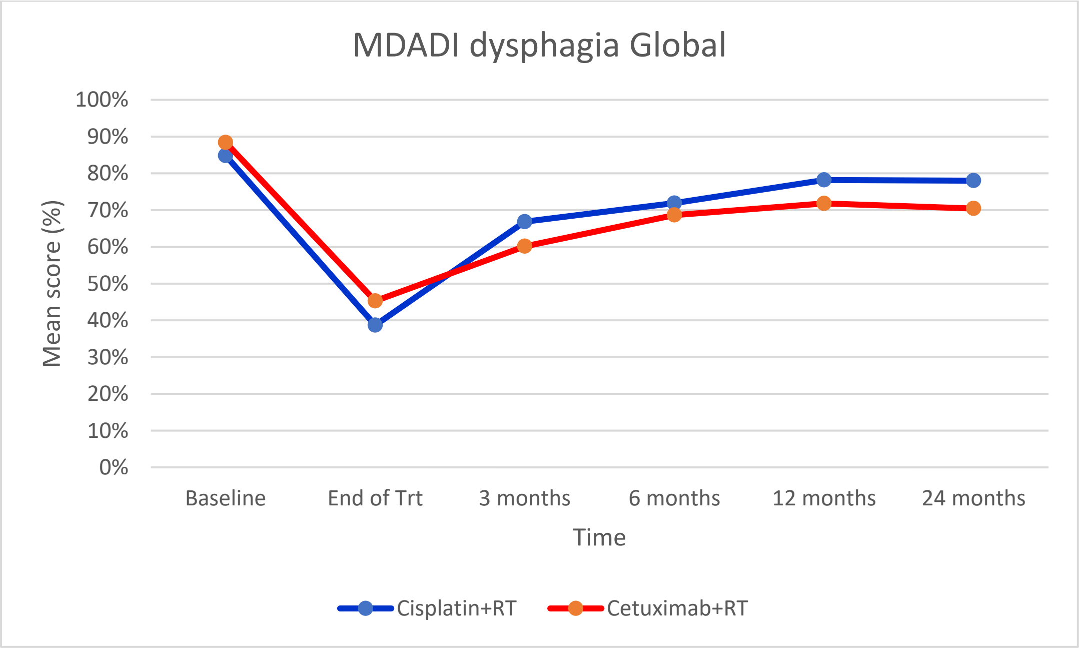

G. MDADI dysphagia emotional

P-value = 0.088

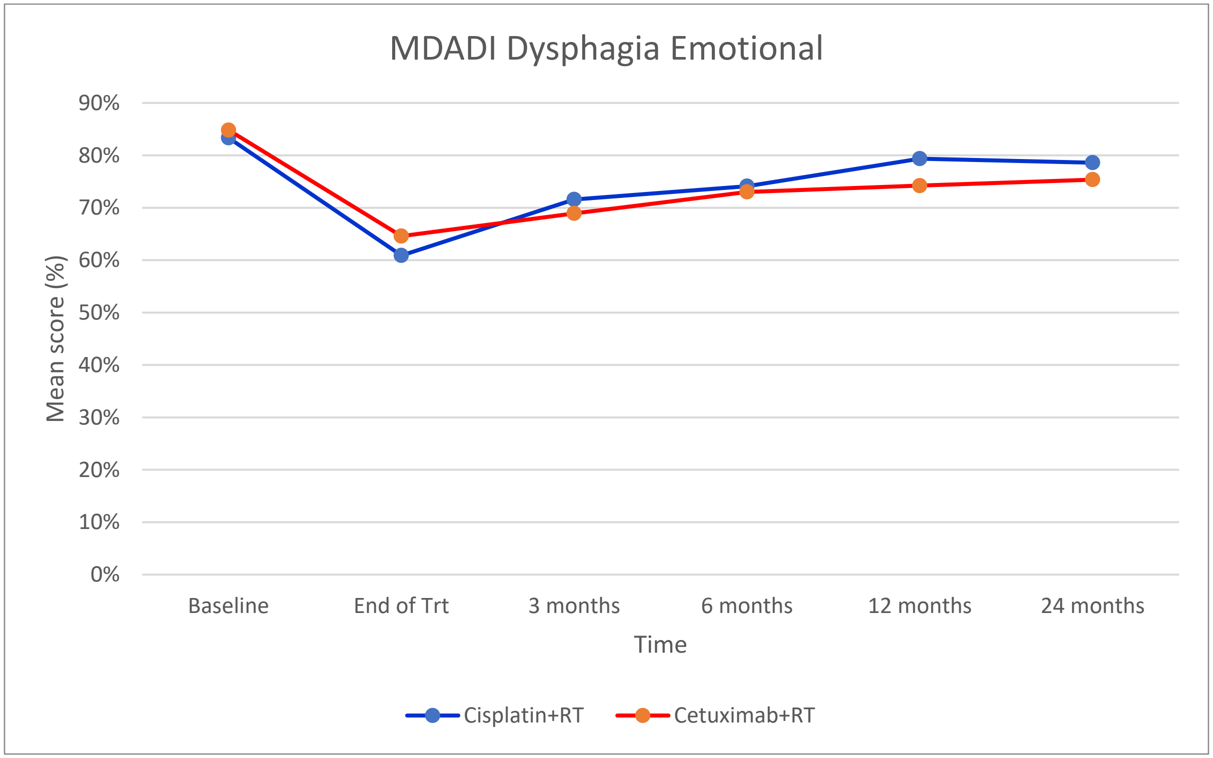

H. MDADI dysphagia functional

P-value = 0.026

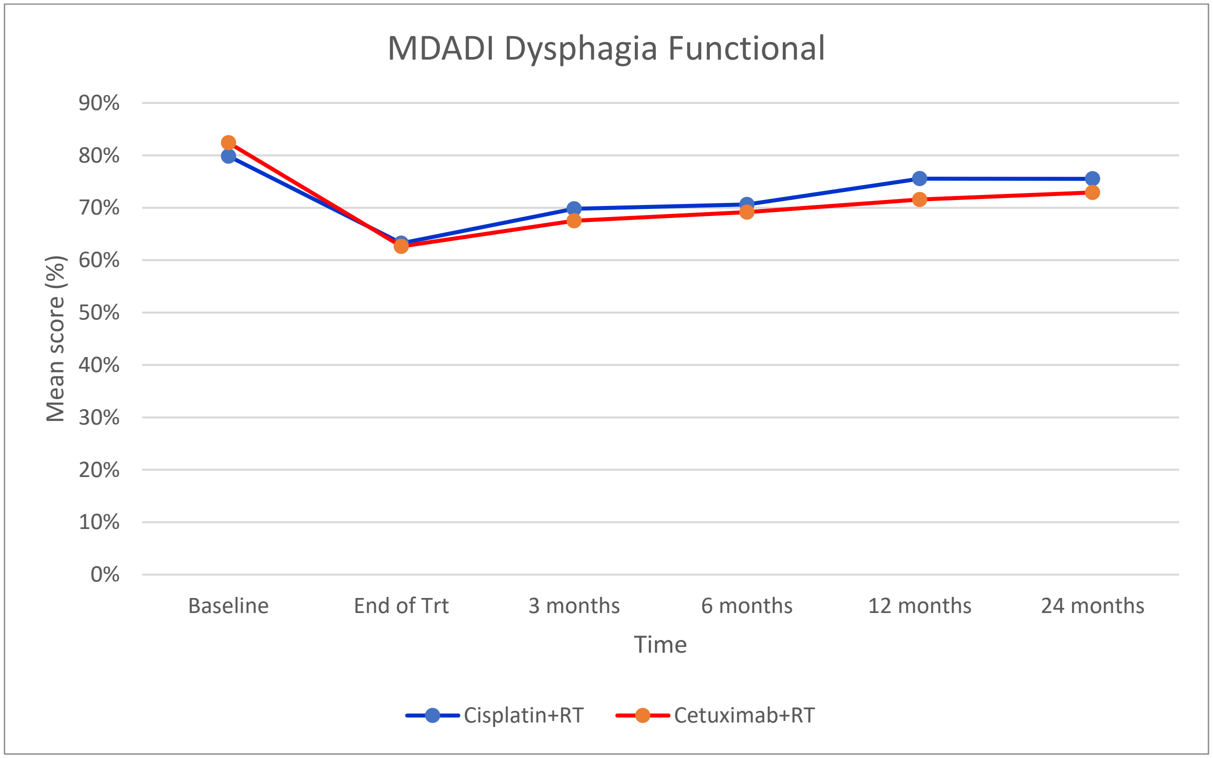

I. MDADI dysphagia physical

P-value = 0.079

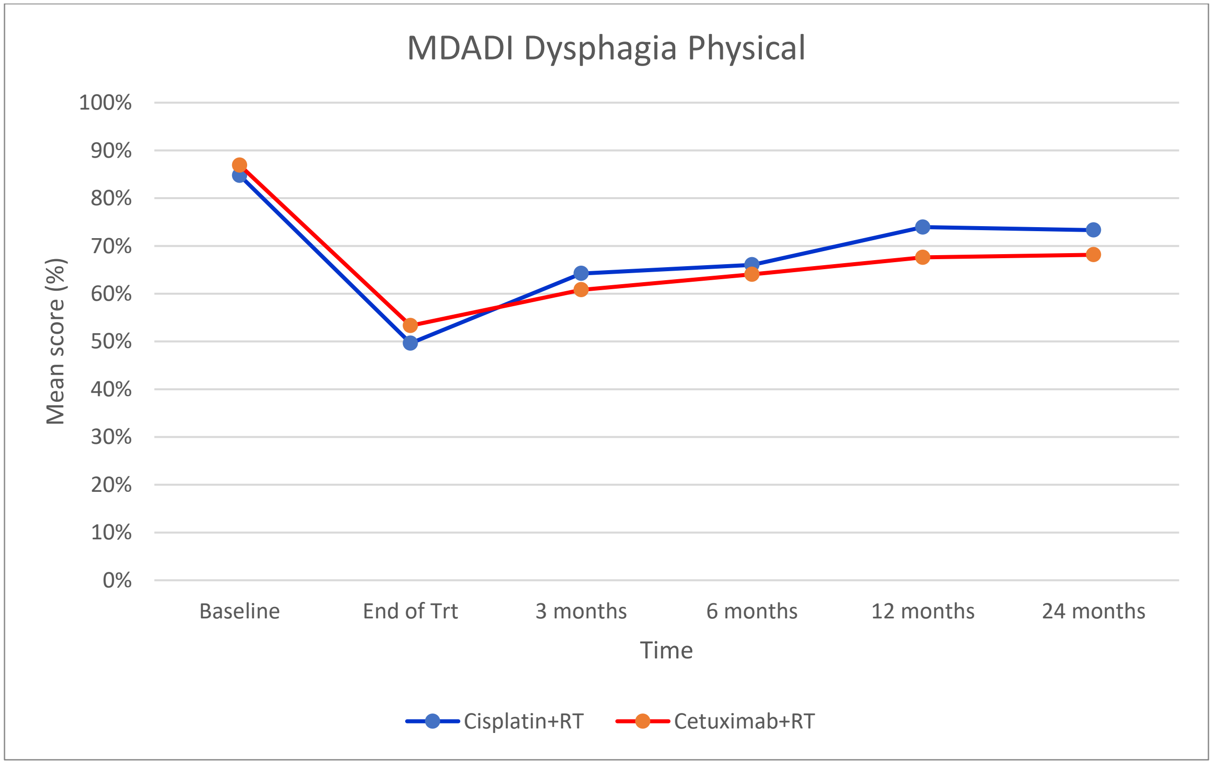

J. MDADI dysphagia overall function

P-value = 0.042

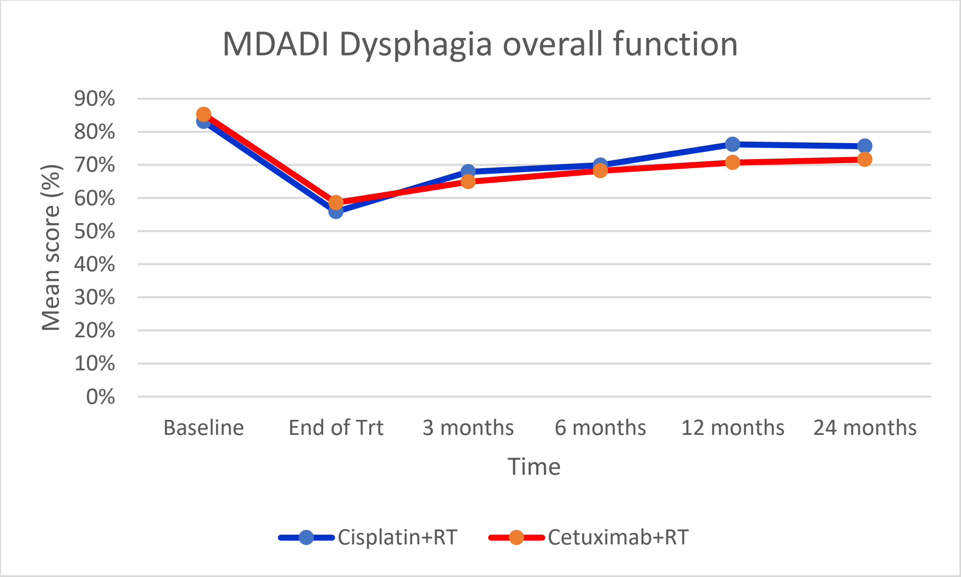

Supplementary References

1. Trotti, A., Pajak, T. F., Gwede, C. K., Paulus, R., Cooper, J., Forastiere, A., et al. (2007). TAME: development of a new method for summarising adverse events of cancer treatment by the Radiation Therapy Oncology Group. The Lancet Oncology, 8(7), 613–624.

Data sharing statement:

Deidentified participant data, and data dictionary will be available along with study protocol and statistical analysis plan; from 01 Jan 2020 onwards. Please write to M.L.Dalby@warwick.ac.uk

1. The final dataset will include basic demographics, tumour characteristics, treatment detail, survival and quality of life and toxicity outcomes. Data will not include name, address, hospital number or date of birth, or any other identifying data.

2. The data will be accompanied by metadata which gives a complete explanation of the data fields, the definition, the standards used such as TNM staging, and the units used.

3. The data will be shared through custodianship by the principle investigator. A data access committee will be convened and will comprise of the principle investigator and two other co investigators. They will be responsible for assessing requests for data sharing on granting access. The data management committee will be responsible to the steering committee and requests for appeals will be made directly to the Trial Steering Committee.

4. The process for requesting data sharing will be as follows:

- *The requestor will complete a two page proforma requiring name and contact details of requestor, the objectives of the study, the methodology, the expected outcome, the statistical analysis plan, whether the project will be a collaboration with the DeESCALaTE study organisers or will only acknowledge the study and its organisers, ethical committee approval, funding and peer review details. The data sharing committee will meet at least twice a year to consider these requests. Urgent requests may be considered in between these meetings.*
- *In the event of a declined application, the requestors may lodge an appeal with the trial Steering Committee Chairperson.*

5. The dataset will be stored with the principle investigator at the Institute of Head and Neck Studies and Education in the long-term. The data will be available for public release from the time of publication of the main results of the study. Prior to that access of the data may be considered in specific circumstances. After five years of publication of the result, the data may then be lodged with a data archiving facility.

6. Requestors who are granted access to the data will be required to complete a data sharing agreement which is based upon the principles, content and format published by the NCRI at http://www.ncri.or.uk/default.asp?s=1&p=8&ss=9
